# Completing Circular Bacterial Genomes With Assembly Complexity by Using a Sampling Strategy From a Single MinION Run With Barcoding

**DOI:** 10.3389/fmicb.2019.02068

**Published:** 2019-09-04

**Authors:** Yu-Chieh Liao, Hung-Wei Cheng, Han-Chieh Wu, Shu-Chen Kuo, Tsai-Ling Yang Lauderdale, Feng-Jui Chen

**Affiliations:** ^1^Institute of Population Health Sciences, National Health Research Institutes, Zhunan, Taiwan; ^2^National Institute of Infectious Diseases and Vaccinology, National Health Research Institutes, Zhunan, Taiwan

**Keywords:** bacterial genome, *de novo* assembly, MinION sequencing, one-stop analysis, assembly complexity

## Abstract

The Oxford Nanopore MinION is an affordable and portable DNA sequencer that can produce very long reads (tens of kilobase pairs), which enable *de novo* bacterial genome assembly. Although many algorithms and tools have been developed for base calling, read mapping, *de novo* assembly, and polishing, an automated pipeline is not available for one-stop analysis for circular bacterial genome reconstruction. In this paper, we present the pipeline CCBGpipe for completing circular bacterial genomes. Raw current signals are demultiplexed and base called to generate sequencing data. Sequencing reads are *de novo* assembled several times by using a sampling strategy to produce circular contigs that have a sequence in common between their start and end. The circular contigs are polished by using raw signals and sequencing reads; then, duplicated sequences are removed to form a linear representation of circular sequences. The circularized contigs are finally rearranged to start at the start position of *dnaA*/*repA* or a replication origin based on the GC skew. CCBGpipe implemented in Python is available at https://github.com/jade-nhri/CCBGpipe. Using sequencing data produced from a single MinION run, we obtained 48 circular sequences, comprising 12 chromosomes and 36 plasmids of 12 bacteria, including *Acinetobacter nosocomialis*, *Acinetobacter pittii*, and *Staphylococcus aureus*. With adequate quantities of sequencing reads (80×), CCBGpipe can provide a complete and automated assembly of circular bacterial genomes.

## Introduction

Illumina sequencing is routinely used to study microbial genomics because of its low cost and the high accuracy of the sequence reads generated. However, the *de novo* assembly of short reads (100–300 base pairs) results in fragmented assemblies because repetitive sequences in bacterial genomes are invariably longer than the length of a short read and the span of paired-end reads. Although accurate draft assemblies can provide some information for comparative analyses, determining the complete genome sequence is still essential. For instance, antimicrobial resistance regions are often flanked by repetitive insertion sequences; in such a case, from an incomplete short-read assembly, it would be impossible to determine whether resistance regions are present in chromosomes or plasmids.

In the past few years, long-read sequencing technologies have been developed by Pacific Biosciences (PacBio) and Oxford Nanopore Technologies (ONT) (Ameur et al., 2018). PacBio and ONT technologies can generate long reads in tens of kilobase pairs, thus making it possible to obtain a complete assembly (Koren and Phillippy, 2014; Liao et al., 2015; Bayliss et al., 2017; Wick et al., 2017a, b; Bainomugisa et al., 2018; Li et al., 2018; Schmid et al., 2018). Compared with the PacBio technology, the ONT MinION is affordable and portable and enables real-time analysis, which render it more attractive for in-field and clinical deployment (Jain et al., 2016; Ameur et al., 2018). The applications of the ONT MinION range from microbial genome assembly to cancer variant discovery and transcript isoform identification (Magi et al., 2017).

Bayliss et al. (2017) demonstrated that multiplexed MinION reads in combination with short reads could complete the genome sequence of *Staphylococcus aureus*. Wick et al. (2017a, b) developed a hybrid assembler, Unicycler, to complete the genome sequences of 12 *Klebsiella pneumoniae* isolates on a single MinION flow cell along with previously available Illumina reads. However, when short-read data are not available, MinION long reads can be used to obtain complete microbial genomes in a rapid and cost-effective manner. Li et al. (2018) obtained the complete sequences of 20 multidrug resistance-encoding plasmids from a barcoded MinION flow cell and demonstrated that long-read assembled plasmids possess high-quality skeletons with correct arrangements of various mobile elements. Long-read assemblers, including Canu (Koren et al., 2017), Flye (Kolmogorov et al., 2018), HINGE (Kamath et al., 2017), and miniasm (Li, 2016), have been developed for *de novo* assembly and can produce a single contig per DNA molecule for some bacterial genomes. Correct completion and circularization of these molecules are essential if they are to be used for monitoring antimicrobial resistance transmission. However, few tools (e.g., Circlator and Unicycler) are available that can automatically produce complete circular DNA structures of bacterial chromosomes and plasmids (Hunt et al., 2015; Wick et al., 2017b). With the emergence of MinION long-read sequencing for complex bacteria, the genomes of *Fusobacterium nucleatum*, *Pseudomonas koreensis*, and *Mycobacterium tuberculosis* harboring long repeats could be completed (Bainomugisa et al., 2018; Schmid et al., 2018; Todd et al., 2018). Nevertheless, a major bottleneck is to analyze nanopore data because many bioinformatics tools are required to be implemented in the five steps of the genome assembly pipeline (i.e., base calling, overlap finding, assembly, read mapping, and polishing) (Magi et al., 2017; Senol Cali et al., 2018). Therefore, we aimed to develop a pipeline and to deploy required tools in a Docker image for one-stop analysis for the reconstruction of a circular bacterial genome by using MinION data. For general audiences who would like to obtain complete bacterial genomes using MinION but are not familiar with bioinformatics skills, we have created a GitHub page^1^ to described how to install and how to run our pipeline.

## Materials and Methods

### DNA Extraction

DNA samples of three *Acinetobacter nosocomialis*, five *A. pittii*, and four *S. aureus* isolates from the Taiwan Surveillance of Antimicrobial Resistance (TSAR) (Ho et al., 1999) were used for the present study. The isolates were recovered from clinical samples taken as part of standard care and the TSAR project was approved by the Research Ethics Committee of the National Health Research Institutes (EC960205, EC1010602-E, EC1030406-E, and EC1050606-E). DNA was extracted using the DNeasy Blood and Tissue Kit (Qiagen) according to extraction protocols provided for gram-positive (*S. aureus*) and gram-negative (*A. nosocomialis* and *A. pittii*) bacteria. In addition, 25 μL of lysostaphin (5 mg/mL) and 2 μL of RNase A (1 mg/mL) were added to 180 μL of an enzymatic lysis buffer for the extraction of DNA from *S. aureus*.

### MinION Library Preparation and Sequencing

Using the ligation methodology, a nanopore sequencing library was constructed using the ligation sequencing kit 1D (SQK-LSK108) and the native barcoding kit (EXP-NBD103) for 12 samples. Briefly, genomic DNA was fragmented into 10 kbp by using g-TUBE (Covaris). Fragmented DNA was repaired and dA-tailed using the NEBNext FFPE DNA Repair Mix and NEBNext Ultra II End Repair/dA-Tailing Module (New England BioLabs). An individual barcode was added to dA-tailed DNA by using the NEB Blunt/TA Ligase Master Mix (New England BioLabs). Each barcoded DNA was pooled in equimolar amounts, and an adaptor was attached using the NEBNext Quick Ligation Module (New England BioLabs). The library was loaded into the SpotON flowcell R9.5 (FLO-MIN107), and sequencing script NC_48Hr_Sequencing_FLO-MIN107_SQK_LSK108 was executed on MinKNOW (V1.7.14).

### Data Analysis

**FIGURE 1:**
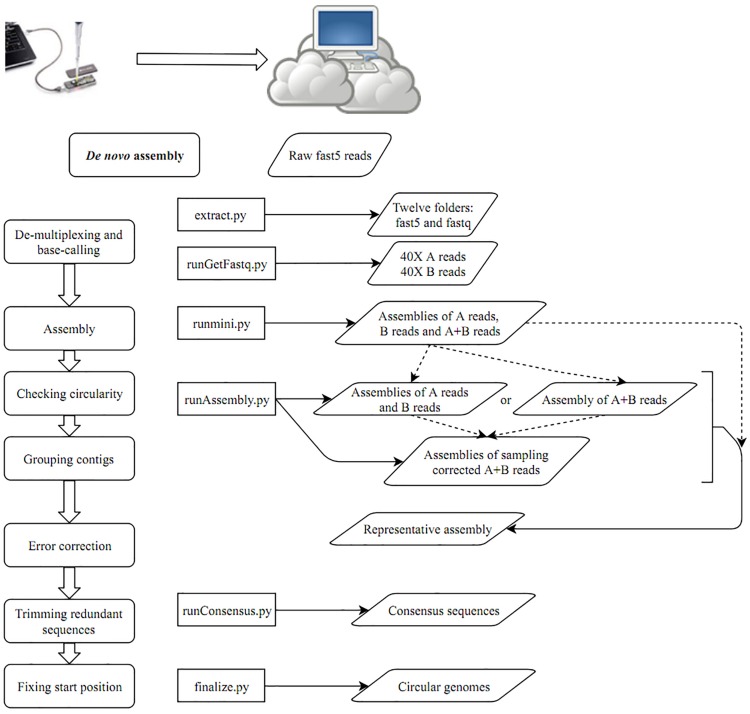
A schematic workflow of CCBGpipe.

After building a Docker image from the Docker file (Supplementary Data), the image was run in a Docker container. A schematic workflow of our proposed pipeline CCBGpipe is shown in Figure 1. A folder containing raw fast5 files was input to extract.py for demultiplexing and base calling with Albacore 2.1.7 (a basecaller released by ONT) to produce an output folder containing 12 barcode folders. The output folder contains 12 barcode folders and 12 sequencing summary files. Each barcode folder contains a fastq file (joinedreads.fastq), an assembly file (assembly.fa), and subfolders containing 4000 fast5 files. The assembly file was produced by minimap2 (Li, 2018) and miniasm (Li, 2016) with the fastq file. The path of the output folder generated by extract.py was input to runGetFastq.py to generate 12 additional barcode folders, with each folder containing 40× long-length reads with quality higher than that in the first quantile (readA.fastq), 40× high-quality reads with length longer than that in the first quantile (readsB.fastq), and concatenated reads (reads.fastq) based on the estimated genome size of the assembly file. Minimap2 and miniasm were used to assemble the three separate read sets to produce three assemblies (assemblyA.fa, assemblyB.fa, and assembly.fa) by using runmini.py. With the runmini-assembled files, an estimated genome size was obtained and used by runAssembly.py to utilize Canu v1.6 (Koren et al., 2017) for the subsequent assembly. Either assemblies of A and B reads or an assembly of A + B reads was produced by Canu depending on the numbers of the circular contigs of the three assemblies produced by runmini.py. If one of the numbers of the circular contigs of assemblies of A and B reads is greater than the number of the circular contigs of the assembly of A + B reads, then Canu is used to assemble A and B reads separately to produce two assemblies (canu.A and canu.B), and the corrected reads are combined for the succeeding assembly by using a sampling strategy; otherwise, Canu is used to assemble A + B reads to produce one assembly (canu.). Canu was used to assemble 40× corrected reads sampled from the combined corrected A and B reads or the corrected A + B reads five times. Each assembly produced by Canu (canu.contigs.fasta) was checked for circularity and the presence of zero depth in misassemblies by using Nucmer (Kurtz et al., 2004) and GraphMap (Sovic et al., 2016), respectively, to prepare a file containing circular and zero-depth-free contigs (cirseqN.fa). By comparing the file size of cirseqN.fa with that of the assembly (assembly.fa) obtained from runmini.py, the number of successful assemblies (i.e., cirseqN.fa > 0.95^∗^assembly.fa) was counted. If this number reaches three, the sampling strategy is terminated early; otherwise, this process remains functional. All circular contigs produced by Canu were concatenated into a file named allcir.fa. All-vs-all alignment of allcir.fa was performed using Nucmer to filter pair alignments between circular contigs with an alignment rate of ≥0.2, an aligned length of ≥2500 bp, and an identity of >0.98. The pair alignments were used as connected components in an undirected graph and then were analyzed using NetworkX (a Python package) to generate connected components in groups. One contig with the longest length among each group was selected as a representative contig to form a representative assembly (canu.cir.fa). If the file size of the representative contig is smaller than that of the miniasm assembly, then the miniasm assembly is polished by Racon (Vaser et al., 2017) and the Racon-polished assembly is then split into 500-kbp-long synthetic reads with an overlap of 10 kbp. These synthetic long reads are combined with 40× sampling reads for Canu assembly for an extra five times. Similarly, the number of successful assemblies was counted. If this number reaches three, the sampling strategy is terminated early; otherwise, this process remains functional until the tenth run. After removing contigs with zero depth and concatenating all circular contigs, representative contigs were selected (fpseq.fa). Nanopolish (Loman et al., 2015) and Racon were used iteratively to polish the representative assembly by running runConsensus.py for consensus sequence generation (conseqs.fasta). Finally, the redundant ends of consensus sequences were trimmed, and circular sequences were rearranged to begin at *dnaA*/*repA* or a position with the minimum value of the GC skew by finalize.py. The scripts used in this study are available at https://github.com/jade-nhri/CCBGpipe, and the usage of CCBGpipe is described in Supplementary Data.

## Results

### MinION Sequencing Data

The ONT MinION sequencing run generated 1,940,879 fast5 reads. After demultiplexing and base calling using Albacore 2.1.7, a total of 991,300 reads (more than 6 Gbp) were obtained for the 12 barcoded samples. As shown in Table 1, the sequencing amount of each sample ranged from 326 Mbp (barcode01) to 696 Mbp (barcode09), and the minimum and maximum sequencing depths were 79× (barcode07) and 241× (barcode09), respectively. Among the 12 samples, the sequence reads and the genomes sequences of the 8 *Acinetobacter* strains (barcode01-barcode08) were released and published (Chen et al., 2019).

**TABLE 1:** Sequencing statistics and assembly information.

**Barcode**	**01**	**02**	**03**	**04**	**05**	**06**	**07**	**08**	**09**	**10**	**11**	**12**

**Organism**	***A. nosocomialis***	***A. pittii***	***A. pittii***	***A. pittii***	***A. pittii***	***A. pittii***	***A. nosocomialis***	***A. nosocomialis***	***S. aureus***	***S. aureus***	***S. aureus***	***S. aureus***
Strain	2012C01-137	2014S07-126	2010C01-170	2012N21-164	2012N08-034	2014N21-145	2010S01-197	2014N23-120				
Biosample^∗^	SAMN09069669	SAMN09069676	SAMN09069679	SAMN09069675	SAMN09069678	SAMN09069674	SAMN09069668	SAMN09069671				
No. of reads	68531	84973	99127	65317	81740	109519	59840	66798	103544	102244	81094	68573
Total bases (Mbp)	326	597	537	450	461	652	336	385	696	646	564	443
Mean length (bp)	4757	7024	5402	6886	5642	5954	5617	5761	6717	6317	6955	6467
Max. length (bp)	58035	89795	74822	78328	65545	55832	51710	54864				
SRA run^∗^	SRR7119551	SRR7119558	SRR7119559	SRR7119553	SRR7119560	SRR7119554	SRR7119552	SRR7119549				
Assembly	3,865,154	3,900,436	4,176,213	3,907,768	3,866,831	3,854,880	4,231,974	4,037,158	2,880,969	2,866,475	2,879,007	2,907,760
	91,208	284,051		97,329	15,039	323,995	134,185	158,983	27,088	27,090	27,085	27,002
	72,978	96,775		73,223	8,159	72,034	92,044	79,057	3,122	3,120	3,118	3,127
	8,080	32,766		12,096	4,340	9,166	6,077	39,960				
		9,207		6,632	3,867		5,449	5,097				
							3,860					
							2,727					

*^∗^The NCBI biosample and SRA accessions.*

### Circular Assemblies

Using the CCBGpipe workflow (Figure 1) described in the Data analysis section, circular chromosome and plasmid sequences were produced for each sample, with a total of 48 complete sequences (Table 1). Barcode01–barcode08 denote *A. nosocomialis* and *A. pittii* samples, and barcode09–barcode12 denote *S. aureus* samples. Among *Acinetobacter* samples, barcode03 contains one chromosome, whereas barcode07 contains one chromosome, two large plasmids (92 and 134 kbp), and four small plasmids; other samples have three or four plasmids. All four *S. aureus* samples have one chromosome (2.8–2.9 Mbp), one large plasmid (∼27 kbp), and one small plasmid (3 kbp). These 48 sequences were all manually examined with Tablet (Milne et al., 2013) to confirm the uniformity and continuity of sequencing coverage.

### Comparison of Assemblers

**FIGURE 2:**
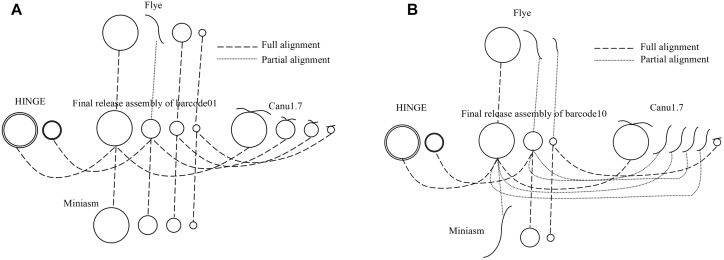
A schematic relationships between assemblies and final release assemblies for **(A)** barcode01 and **(B)** barcode10. Full alignment represents a near-perfect correlation between a circular contig and a circular sequence, partial alignment represents a partial correlation between a linear contig and a circular sequence.

All the sequencing reads of each barcode were *de novo* assembled using Canu (v1.7), Flye (2.3.3-g47cdd0b), HINGE, and miniasm (0.2-r168-dirty), for which default settings were applied (commands are shown in Supplementary Data). Because Canu and Flye require genome size as an input parameter, the genome size obtained using miniasm was used for Canu and Flye. Although Canu outputs “suggestCircular=yes” in the header line for circular sequences, we examined circularity ourselves. Flye provides assembly_info.txt to indicate whether a sequence is circular. HINGE produces an assembly along a graph, from which a circular path can be observed for a circular sequence. Miniasm outputs an assembly graph containing unitigs with “c” and “l” suffixes to represent circular and linear sequences, respectively. The number of circular contigs for each assembler is summarized in Table 2. The relationships between assemblies and the final release assemblies are shown in Figure 2, illustrating how we determine the numbers in Table 2. Take barcode01 as an example, Canu1.7 assemble four contigs with overlapping sequences at contig ends and each of the four circular contigs is near-perfect correlated to the final release assembly; Flye assemble four contigs and one linear contig is partial correlated to a plasmid. In addition to provide the full descriptions about full alignment and partial alignment in Supplementary Data, genome size and number of contigs produced by these assemblers are also listed in Supplementary Data. Among the four assemblers, miniasm produced as many as 37 circular contigs (out of 52 contigs) for 12 samples, but the assemblies were less accurate and lacked an error correction stage. Except for miniasm, the numbers of circular sequences produced by the other three assemblers, namely Canu, Flye and HINGE, were comparable: 27–31 circular sequences. As shown in Table 2, Canu and Miniasm produce the most 4 circular sequences for barcode01, Canu and HINGE produce one circular chromosome sequence for barcode03, Miniasm produces 5 circular sequences for barcode05, and Flye produce 3 circular sequences for barcode12, which suggests that each of the assemblers has its own merits in assembling various sequencing reads. However, it is unrealistic to exhaustively try various assemblers to produce circular sequences as many as possible. We therefore introduce a sampling strategy of reads on Minasm and Canu to see how they work.

**TABLE 2:** Circular sequences deduced by assemblers.

	**Canu1.7**	**Flye**	**HINGE**	**Miniasm**
Barcode01	**1 chromosome 3 plasmids**	1 chromosome 2 plasmids	1 chromosome 1 plasmid	**1 chromosome 3 plasmids**
Barcode02	3 plasmids	1 chromosome 2 plasmids	1 chromosome 2 plasmids	**1 chromosome 4 plasmids**
Barcode03	**1 chromosome**	0	**1 chromosome**	0
Barcode04	1 chromosome 1 plasmid	1 chromosome 2 plasmids	1 chromosome	1 chromosome 3 plasmids
Barcode05	3 plasmids	1 chromosome 1 plasmid	1 chromosome 3 plasmids	**1 chromosome 4 plasmids**
Barcode06	1 chromosome 2 plasmids	1 chromosome 2 plasmids	1 chromosome 2 plasmids	3 plasmids
Barcode07	1 chromosome 3 plasmids	1 chromosome 5 plasmids	2 plasmids	3 plasmids
Barcode08	2 plasmids	1 chromosome 2 plasmids	1 chromosome 2 plasmids	4 plasmids
Barcode09	**1 chromosome 2 plasmids**	1 chromosome 1 plasmid	1 chromosome 1 plasmid	2 plasmids
Barcode10	1 chromosome 1 plasmid	1 chromosome	1 chromosome 1 plasmid	2 plasmids
Barcode11	0	1 chromosome 1 plasmid	1 chromosome 1 plasmid	**1 chromosome 2 plasmids**
Barcode12	1 chromosome 1 plasmid	**1 chromosome 2 plasmids**	1 chromosome 1 plasmid	1 chromosome 1 plasmid
Total	28	31	27	37

*Complete genomes are shown in bold.*

### Miniasm Assemblies

Miniasm, a fast assembler, was used to assemble all fastq reads and subsets of reads, followed by read overlap detection with minimap2 (Li, 2018). As listed in Table 3, miniasm assembled all the reads of each strain into as many as 37 circular sequences (suffixed with “c” in the output assembly), but it assembled only 25 circular sequences when 40× long-length reads (A^∗^ reads) were input. Miniasm produced similar assemblies — 35 circular sequences for A^∗^ + B^∗^ reads and 38 for A + B reads — when a total of 80× reads (high-quality reads and long-length reads) were input. A^∗^ and B^∗^ are reads ordered by length and quality, respectively, without considering the minimal quality and length, and some of them overlap. Forty-fold sampling reads from the read sets (all reads, A^∗^ + B^∗^ and A + B) were separately produced and assembled 10 times by using miniasm. Because the CCBGpipe-assembled sequences (Table 1) were all manually checked and confirmed as complete circular sequences, we took these 48 sequences as benchmark in the following comparisons. Among the 30-times assemblies of sampling reads, complete circular sequences were found in barcode01–barcode06 and barcode12, but small plasmids (3 kbp) were often missing in barcode09–barcode12 (Table 3 and Supplementary Figure 1). Although the sampling strategy applied to miniasm could provide 42 circular sequences at the most (Supplementary Figure 1), six sequences were missing as compared with the 48 sequences in Table 1, namely two chromosomal sequences (more than 4 Mbp), two large plasmids (92 and 158 kbp), and one small plasmid (3.8 kbp) in barcode07 and barcode08 and one small plasmid (3 kbp) in barcode11.

**TABLE 3:** Number of circular contigs produced by miniasm and Canu with different subsets of reads.

**Barcode number**	**01**	**02**	**03**	**04**	**05**	**06**	**07**	**08**	**09**	**10**	**11**	**12**	**Sum**

**No. of sequences**	**4**	**5**	**1**	**5**	**5**	**4**	**7**	**5**	**3**	**3**	**3**	**3**	**48**

**Miniasm**	**No. of circular contigs**												
All reads	**4**	**5**	0	4	**5**	3	3	4	2	2	**3**	2	37
A^∗^ reads	3	3	0	3	2	3	1	2	2	2	2	2	25
B^∗^ reads	3	4	0	**5**	4	**4**	1	1	**3**	1	1	**3**	30
A^∗^ + B^∗^ reads	**4**	**5**	0	4	**5**	**4**	3	3	2	1	2	2	35
A + B reads	4	5	0	4	5	4	3	4	3	2	2	2	38
40× sampling^#^ all	**4**	4	**1**	**5**	**5**	**4**	4	3	**3**	**3**	2	**3**	41
40× sampling^#^ A^∗^ + B^∗^	**4**	**5**	**1**	**5**	**5**	**4**	4	2	2	2	2	**3**	39
40× sampling^#^ A + B	**4**	4	**1**	**5**	**5**	**4**	4	3	2	2	2	**3**	39
**Canu1.6**													
A^∗^ reads	2	3	**1**	2	2	3	2	1	2	1	0	1	20
B^∗^ reads	2	2	0	3	2	2	4	2	2	1	2	2	24
A reads	3	2	**1**	2	1	2	0	1	0	0	0	0	12
B reads	3	4	0	4	2	2	3	2	2	1	**3**	2	28
A + B reads	**4**	3	**1**	3	2	2	6	3	2	2	2	2	32
**Canu1.7**													
All reads	**4**	3	**1**	2	3	3	4	2	**3**	2	0	2	28
A reads	3	2	**1**	2	1	2	0	1	0	0	0	1	13
B reads	3	4	0	4	1	2	3	2	2	2	2	**3**	28
A + B reads	3	3	**1**	1	4	3	6	3	**3**	1	2	2	32

*A^∗^: 40× long-length reads, B^∗^: 40× high-quality reads. A: 40× long-length reads with quality higher than that in the first quantile, B: the remaining 40× high-quality reads with length longer than that in the first quantile. ^#^Forty-fold sampling reads from the read sets (all reads, A^∗^ + B^∗^ and A + B) were separately produced and assembled ten times. The highest number of circular contigs produced in different datasets were highlighted in bold.*

### Canu Assemblies

Two versions of Canu (v1.6 and v1.7) were used in this study. Because a sequencing depth between 30× and 60× is recommended in Canu, reads with an accumulated read length of more than 40× genome size estimated on the basis of the miniasm assembly were selected. Assemblies produced by Canu were examined for circularity, and the numbers of circular sequences are listed in Table 3. By default, in Canu v1.6, the longest 40× of input reads is used (based on a specified genome size), which is equivalent to inputting A^∗^ reads (40× long-length reads). However, the number of assembled circular sequences obtained from A^∗^ reads is slightly smaller than that obtained from B^∗^ reads (40× high-quality reads): 20 versus 24 (Table 3). Canu v1.6 assembled 40× high-quality reads (B^∗^) into additional 14 small plasmids (11 sequences were smaller than 10 kbp and the other three were 12, 27, and 39 kbp, respectively) but missed six chromosomal sequences and four large plasmids (ranging from 15 kbp to 4.2 Mbp; Supplementary Figure 2), in comparison with assembling 40× long-length reads (A^∗^). Canu v1.7 improved support for plasmids through read rescue, such that it took not only the 40× longest reads but also short reads from plasmids by selecting poorly represented sequences as input. As shown in Tables 2, 3, 28 circular sequences were assembled using Canu v1.7 with default settings from all reads. Therefore, complete circular genomes were obtained for three strains: barcode01, barcode03, and barcode09. Nevertheless, Canu tended to miss substantial circular genomes when long-read sets (Table 3) were each assembled. As depicted in Table 3 and Supplementary Figure 2, similar sequences were assembled by Canu v1.6 and v1.7 from A or B reads, but considerable differences were observed between Canu v1.6 assemblies obtained from A/A^∗^ and B/B^∗^ reads, suggesting that the constitute reads of an inputting file determine an assembly. In addition to inputting 40× reads into Canu using default settings, we assembled A + B reads (80×) with corOutCoverage = 1000, which increased the number of circular sequences to 32. Although 32 circular sequences were found the most in single-run assemblies obtained from Canu, a total of 46 circular sequences were observed in the nine runs of Supplementary Figure 2 (only one chromosome in barcode08 and one plasmid in barcode02 are missing). These results indicate that the sampling strategy is considerably likely to increase genome completeness.

### Assembly Workflow

On the basis of the aforementioned results, we developed a pipeline for completing circular bacterial genomes; the workflow is summarized as follows. When MinION started to generate fast5 reads, files were transferred to a separate Linux server for demultiplexing and base calling by using Albacore 2.1.7 by running extract.py. Accordingly, 12 folders containing demultiplexed fast5 reads and base-called fastq files were prepared (Figure 1). Miniasm (Li, 2016) was used to assemble fastq reads separately for estimating the genome size. To provide adequate long reads for subsequent sampling assembly, we selected 80× reads for analysis. The 40× long-length reads with quality higher than that in the first quantile were selected as A reads, and the remaining 40× high-quality reads with a length longer than that in the first quantile were selected as B reads by running runGetFastq.py. The distributions of read quality and read length can be seen in Supplementary Figure 3. For each barcode, three miniasm assemblies were generated by running runmini.py with A, B, and A + B reads. The numbers of circular contigs assembled by miniasm with A, B, and A + B reads determine the Canu assembly (runAssemble.py): using A and B reads separately or using A + B reads. In addition to producing either one Canu assembly directory (canu.) or two Canu assembly directories (canu.A and canu.B), corrected and trimmed reads in the canu.^∗^directory were used for sampling. The 40× sampling reads from corrected A + B reads were separately produced and also assembled by Canu (specifying “-assemble” in the Canu command) five times. Each contig was examined for overlaps at both ends, suggesting circularity of the sequence. Non-circular contigs were excluded from the subsequent sequence grouping. Because multiple assemblies were obtained by assembling sampled reads, superfluous contigs were removed by grouping sequences with all-to-all alignments. The longest contig was selected as a representative contig for each group. If the file size of the representative contig was smaller than that of the miniasm assembly, then the miniasm assembly was polished using Racon and subsequently split into 500-kbp synthetic long reads with an overlap of 10 kbp. These synthetic ultra-long reads were combined with 40× sampling reads for extra five assemblies. The representative assembly was polished by Nanopolish (Loman et al., 2015) and Racon (Vaser et al., 2017) using runConsensus.py. To prevent the loading of all raw signal files for polishing a specific strain, in running extract.py we have binned the fast5 files into their corresponding folders. After this implementation, the computational time required for running Nanopolish decreased considerably (from more than 8 to 1 h for one iteration). After two iterations of Nanopolish, we performed Racon twice, followed by a final polishing step by using Nanopolish. Subsequently, redundant ends were trimmed out to rearrange the final start position at *dnaA*/*repA* or the replication origin based on the GC skew (finalize.py).

In addition to complete the genome sequences of our 12 samples using CCBGpipe, we utilized our pipeline to run 7 samples of *K. pneumoniae* (in Supplementary Data), whose ONT reads exceeding 80× depth among a set of 12 (Wick et al., 2017a). Wick et al. (2017a) produced 33 circular sequences using Unicycler hybrid assembler with Illumina and ONT reads from these 7 samples, then manually finalized the assemblies to include 36 sequences. By utilizing ONT reads only, CCBGpipe produced 33 circular sequences, which outperformed long-read-only assemblies produced by Unicycler (26 circular sequences) and Canu (16 circular sequences). Please note that when assembling just long reads with Unicycler, it uses miniasm for assembling and couples with multiple rounds of Racon for polishing. In summary, our pipeline produced more complete circular sequences than other assemblers, including Canu, Flye, HINGE, miniasm, and Unicycler.

## Discussion

The development of MinION nanopore sequencing has made it possible to obtain multiple complete plasmid sequences in a single MinION run according to a rapid barcoding protocol (Li et al., 2018). Although the rapid barcoding sequencing kit (SQK-RBK001) can prepare a library within 10 min, it produced total bases less than 500 Mbp. In this study, we used the 1D ligation sequencing kit (SQK-LSK108) to produce nanopore sequencing reads of more than 6 Gbp for a total of 12 samples and successfully assembled them into circular genomes. To the best of our knowledge, this is the first study to complete 12 multiplexing bacterial genomes in a single MinION flow cell run without incorporating any complementary short-read sequencing data. The 48 circular sequences produced by CCBGpipe were manually examined. Nevertheless, one may argue the absence of true benchmarks. We therefore validated the completeness of assemblies produced by CCBGpipe by running the 7 samples in Wick et al. (2017a). The number of circular contigs produced by CCBGpipe is the most compared to the long-read-only assemblies produced by Canu and Unicycler. Besides, it produced the identical number of circular contigs to the Unicycler hybrid assemblies, which suggests that CCBGpipe appears to provide the most complete circular sequences with only long reads.

In terms of the repeat count and maximum repeat length, some species of *Acinetobacter* belong to the most complex microbial genome class III (Koren et al., 2013; Wick et al., 2017a), which comprises a maximum repeat size of more than 7 kbp and requires very long reads (>10 kbp) to resolve genome complexity. Miniasm and Canu are the two assemblers commonly used for nanopore assembly (Senol Cali et al., 2018); however, they could not complete all 48 circular sequences in a single run in the present study. In addition, Flye and HINGE were used to assemble all reads, and they could produce only 31 and 27 circular sequences, respectively. Compared with Canu, the other three long-read assemblers required less computational time but produced less accurate assemblies (see Supplementary Data). The sequence identity to the final release was 99.4, 89.0, 98.0, and 98.0% for Canu, miniasm, Flye, and HINGE, respectively. Vaser et al. (2017) reported that Racon coupled with miniasm enables accurate genome completion and is an order of magnitude faster (Vaser et al., 2017). In this study, we confirmed that the sequence identity to the final release increased from 89.0 to 99.5% when Racon was applied after miniasm. The sampling strategy marginally increased the number of circular sequences from 38 to 41 in miniasm assemblies (Table 2); thus, this strategy was principally implemented in Canu. Although Canu is the most time consuming among the long-read assemblers, with the correction stage, it provides near-identical overlaps at each end of contigs if they represent circular sequences, which facilitates sequence circularization by removing duplicated sequences at contig ends.

Because of the lack of Illumina sequencing reads for polishing the assemblies, we implemented multiple iterations of Racon and Nanopolish for consensus sequence generation. Wick et al. (2019) reported that the maximum accuracy was obtained at 99.6% identity after one round of Nanopolish. With multiple iterations of Racon and Nanopolish in CCBGpipe, the accuracy of long-read-only assemblies should exceed 99.8% identity. In Supplementary Data, we have assessed the accuracy of two *S. aureus* assemblies produced by CCBGpipe to be of 99.87%, and the accuracies for raw MinION reads, long-read-only Unicycler, and Canu assemblies are 90, 99.7, and 99.5%, respectively. Nevertheless, an error rate of 0.2% still impeded the application of nanopore-based sequencing in accurate gene calling, while multilocus sequence typing (MLST) and single nucleotide polymorphisms (SNP) genotyping (Cornelis et al., 2017; Tarumoto et al., 2017; Votintseva et al., 2017) were feasible. When complete genome sequences were available, ResFinder 3.1 (Zankari et al., 2012) was used to identify acquired antimicrobial resistance genes for the 12 beta-lactam-resistant isolates (Table 1). Although three *A. nosocomialis* isolates (barcode01, barcode07, and barcode08) all harbored the *bla*_OXA–__58_ gene in plasmids, the *bla*_OXA–__500_ gene was found in chromosomes in *A. pittii* (barcode02, and barcode04–06). In addition, multiple carbapenemases were found in *A. pittii*; *bla*_*OXA–*__58_ and *bla*_IMP–__1_ were present in a plasmid of barcode02. A detailed study about carbapenem resistance in *A. nosocomialis* and *A. pittii* based on the complete genomes has been published (Chen et al., 2019). In *S. aureus* (barcode09–barcode12), beta-lactamases are encoded by the *blaZ* gene, which is located in the large plasmid (27 kbp), and the *mecA* gene is located in the chromosome. Our results provide compelling evidence for the necessity of assembling the whole genome rather than focusing on plasmids, and they suggest that our pipeline can provide the most complete circular sequences. However, our study has some limitations. First, Canu and Nanopolish both required extensive computation, and approximately 1 day was required on a 16-core server with 96 GB of memory to complete one barcoded bacterial genome. Second, the ONT-only final assembly had an error rate of 0.2%. Although Canu requires a low coverage of 20×, our pipeline prefers the coverage of 80× for assembling all pieces of DNA including chromosomes plus plasmids. Third, in lacking of the true numbers and sizes of plasmids for the 12 strains, we may miss several plasmid sequences which can only be confirmed by further experiments. Over the past 2 years, the sequencing quality and throughput of MinION have considerably improved (Magi et al., 2017; Tyler et al., 2018). For example, the rapid barcoding sequencing kit SQK-RBK004 produced more than 5 Gbp [a 10-fold increase compared with the old kit (Li et al., 2018)], and the base caller (Albacore) moved from the hidden Markov model (HMM) to the recurrent neural network (RNN) for accurate base calling using raw signal^2^. Recently, ONT released Guppy to replace Albacore for higher basecall accuracy and speed (Supplementary Data), they also released Medaka for rapid error correction of sequencing data. Therefore, future advances in nanopore technology and computational methods may reduce computational time and increase accuracy.

Senol Cali et al. (2018) conducted a review and analyzed state-of-the-art tools associated with the genome assembly pipeline. After comprehensive analysis, they recommended that Scrappie^3^, minimap, miniasm, and Racon should be used for base calling, read mapping, assembly, and polishing, respectively. All the tools, namely minimap2, GraphMap, Canu, miniasm, Nanopolish, and Racon, were utilized in our pipeline, except for Scrappie. Albacore, ONT’s official base caller, was used for base calling and has been recommended to be the best base caller after a thorough comparison by Wick et al. (2019)^4^. In summary, we presented a pipeline through which an initial assembly can be rapidly obtained using miniasm and a complete final assembly can be produced using Canu coupled with a sampling strategy. This pipeline written in Python can be easily conducted to process raw signals (fast5) produced by MinION and to obtain long-length/high-quality fastq reads, miniasm and Canu assemblies, and complete and high-quality genomes. According to the announcement of ONT, they are targeting, by a variety of method including a new design of nanopore (R10) and a new basecaller (Guppy), a Q-score of 50 then 60 (one error per megabase) for consensus accuracy enhancement. Therefore, we could expect that our CCBGpipe will help bacteriologist to produce highly accurate complete finished genomes by ONT-only long-read sets.

## Data Availability

Publicly available datasets were analyzed in this study. This data can be found here: https://www.ncbi.nlm.nih.gov/bioproject/PRJNA459525.

## Author Contributions

Y-CL and F-JC conceived this project. H-CW conducted the sequencing experiments. Y-CL and H-WC implemented the pipeline. S-CK and T-LL provided the strains. Y-CL, S-CK, T-LL, and F-JC advised and participated in discussion of the study. Y-CL and F-JC drafted the manuscript. All authors read and approved the final version of the manuscript.

## Conflict of Interest Statement

The authors declare that the research was conducted in the absence of any commercial or financial relationships that could be construed as a potential conflict of interest.

## Footnotes

1 https://github.com/jade-nhri/CCBGpipe

2 https://community.nanoporetech.com/

3 https://github.com/nanoporetech/scrappie

4 https://github.com/rrwick/Basecalling-comparison
