# Supplementary material for: Completing Circular Bacterial Genomes With Assembly Complexity by Using a Sampling Strategy From a Single MinION Run With Barcoding
Source: Front Microbiol. 2019 Sep 4;10:2068. doi: 10.3389/fmicb.2019.02068 (PMC6737777; doi:10.3389/fmicb.2019.02068)
Supplement: Supplementary file 1 [file Data_Sheet_1.PDF]

Supplementary Material

1 Supplementary Data

Dockerfile

```
FROM ubuntu:16.04
RUN apt-get update && apt-get install -y \
    wget dos2unix \
    python3 \
    python3-pip \
    python3-setuptools \
    python3-dev \
    cmake unzip
RUN pip3 install networkx
RUN pip3 install pandas
RUN pip3 install pyfastaq

##Download CCGBpipe
WORKDIR /opt
RUN git clone https://github.com/jade-nhri/CCBGpipe.git
WORKDIR /opt/CCBGpipe/CCBGpipe
RUN chmod +x *.py

#albacore 2.1.7
#This software requires the user to download manually!!!

#samtools 1.7
ADD https://github.com/samtools/samtools/releases/download/1.7/samtools-1.7.tar.bz2 /opt
RUN apt-get update && apt-get install -y \
    libncurses-dev \
    apt-file \
    liblzma-dev \
    libz-dev \
    libbz2-dev \
    vim parallel
WORKDIR /opt
RUN tar -xjf /opt/samtools-1.7.tar.bz2
WORKDIR /opt/samtools-1.7
RUN make && make install
WORKDIR /

#bwa BWA-0.7.17
RUN apt-get install -y git
WORKDIR /opt
```

```
RUN git clone https://github.com/lh3/bwa.git
WORKDIR /opt/bwa
RUN make
WORKDIR /

#nanopolish v0.9.0
RUN apt-get update && apt-get install -y python-pip python-dev python-biopython build-essential
python-matplotlib
WORKDIR /opt
RUN git clone --recursive https://github.com/jts/nanopolish.git
WORKDIR /opt/nanopolish
RUN git checkout v0.9.0
RUN make
WORKDIR /

#canu v1.6
WORKDIR /opt
RUN wget https://github.com/marbl/canu/archive/v1.6.tar.gz
RUN gunzip -dc v1.6.tar.gz | tar -xf -
WORKDIR /opt/canu-1.6/src
RUN make -j 16
WORKDIR /

#MUMmer 3.23
WORKDIR /opt
RUN wget https://sourceforge.net/projects/mummer/files/mummer/3.23/MUMmer3.23.tar.gz
RUN tar -zxvf MUMmer3.23.tar.gz
WORKDIR /opt/MUMmer3.23
RUN make
RUN make install
WORKDIR /

#Minimap2, miniasm-0.2
WORKDIR /opt
RUN curl -L https://github.com/lh3/minimap2/releases/download/v2.10/minimap2-2.10_x64-
linux.tar.bz2  | tar -jxvf -
RUN wget https://github.com/lh3/miniasm/archive/v0.2.tar.gz \
   && tar -xzf v0.2.tar.gz \
   && (cd /opt/miniasm-0.2 && make) \
   && rm v0.2.tar.gz
WORKDIR /

#Racon1.1.1
WORKDIR /opt
RUN wget https://github.com/isovic/racon/releases/download/1.1.1/racon-v1.1.1.tar.gz \
   && tar -xzf racon-v1.1.1.tar.gz \
   && (cd /opt/racon-v1.1.1 && cmake -DCMAKE_BUILD_TYPE=Release && make) \
```

```
    && rm racon-v1.1.1.tar.gz
WORKDIR /

#Graphmap v0.3.0
WORKDIR /opt
RUN git clone https://github.com/isovic/graphmap.git
WORKDIR /opt/graphmap
RUN make modules && make
WORKDIR /

#set path
ENV PATH $PATH:/opt:/opt/CCBGpipe/CCBGpipe:/opt/samtools-
1.7/bin:/opt/bwa:/opt/nanopolish:/opt/canu-1.6/Linux-amd64/bin:/opt/MUMmer3.23:/opt/minimap2-
2.10_x64-linux/:/opt/miniasm-0.2:/opt/racon-v1.1.1/bin:/opt/graphmap/bin/Linux-x64
```

Usage of CCBGpipe

A folder named “raw_reads”: contains the flowing folders

Name	Date modified	Type
20171016_0840_20171016	2017/11/1 下午 12:55	File folder
20171016_0847_20171016	2017/10/23 下午 05:39	File folder
20171017_0109_20171016	2017/10/23 下午 05:41	File folder
20171017_0116_20171016	2017/10/23 下午 05:42	File folder
20171018_0110_20171016	2017/10/23 下午 05:42	File folder
20171018_0117_20171016	2018/8/14 下午 03:27	File folder

Since the file size of raw reads for a MinION single flow cell are too large, we provide raw reads of a single barcode for testing.

https://drive.google.com/uc?export=download&confirm=TxIT&id=1e-xYLDEEzi8UqRf30KVTymmHNxr_te7P

```
5387428158 Apr 17 03:35 barcode01.tar.gz
```

wget --load-cookies /tmp/cookies.txt "https://docs.google.com/uc?export=download&confirm=$(wget --quiet --save-cookies /tmp/cookies.txt --keep-session-cookies --no-check-certificate 'https://docs.google.com/uc?export=download&id=1e-xYLDEEzi8UqRf30KVTymmHNxr_te7P' -O- | sed -rn 's/.*confirm=([0-9A-Za-z_]+).*/\1\n/p')&id=1e-xYLDEEzi8UqRf30KVTymmHNxr_te7P" -O barcode01.tar.gz && rm -rf /tmp/cookies.txt

To extract fastq and fast5 files using extract.py:

```
usage: extract.py [-h] [-flowcell FLOWCELL] [-kit KIT] [-t T] inpath outpath

positional arguments:
  inpath               the path to raw_reads folder
  outpath              an output folder

optional arguments:
  -h, --help           show this help message and exit
  -flowcell FLOWCELL   flowcell (default=FLO-MIN107)
  -kit KIT             kit (default=SQK-LSK108)
  -t T                 threads (default=60)
```

extract.py raw_reads albacore2.1.7 -flowcell FLO-MIN107 -kit SQK-LSK108 -t 100

You will get a folder named albacore2.1.7, and it contains a fast5 folder.

```
   4096 Apr 17 06:22 ./
   4096 Apr 19 03:39 ../
6512210 Apr 17 06:22 copiedfiles.txt
   4096 Apr 17 06:22 fast5/
      0 Apr 17 06:22 filelist.txt
```

Inside the fast5 folder, there are 12 barcode folders and 12 sequencing summary files:

```
    4096 [illegible]  5  2018 barcode01/
12772631 [illegible]  3  2018 barcode01.txt
    4096 [illegible]  5  2018 barcode02/
15857355 [illegible]  3  2018 barcode02.txt
    4096 [illegible]  5  2018 barcode03/
18479467 [illegible]  3  2018 barcode03.txt
    4096 [illegible]  5  2018 barcode04/
12185340 [illegible]  3  2018 barcode04.txt
    4096 [illegible]  5  2018 barcode05/
15241336 [illegible]  3  2018 barcode05.txt
    4096 [illegible]  5  2018 barcode06/
20426701 [illegible]  3  2018 barcode06.txt
    4096 [illegible]  5  2018 barcode07/
11156870 [illegible]  3  2018 barcode07.txt
    4096 [illegible]  5  2018 barcode08/
12452663 [illegible]  3  2018 barcode08.txt
    4096 [illegible]  5  2018 barcode09/
19322667 [illegible]  3  2018 barcode09.txt
    4096 [illegible]  5  2018 barcode10/
19079531 [illegible]  3  2018 barcode10.txt
    4096 [illegible]  5  2018 barcode11/
15135040 [illegible]  3  2018 barcode11.txt
    4096 [illegible]  5  2018 barcode12/
12794961 [illegible]  3  2018 barcode12.txt
```

Each barcode folder contains fast5 folders (4000 fast5 files included), FASTQ file (joinedreads.fasta) and an assembly.fa (preassembled by minimap2 and miniasm with joinedreads.fastq).

```
     4096 Apr 17 06:22 ./
     4096 Apr 17 06:22 ../
   598016 Apr 17 03:52 0/
   610304 Apr 17 04:00 1/
   606208 Apr 17 05:29 10/
   606208 Apr 17 05:37 11/
   606208 Apr 17 05:45 12/
   598016 Apr 17 05:57 13/
   606208 Apr 17 06:05 14/
   606208 Apr 17 06:17 15/
   454656 Apr 17 06:22 16/
   614400 Apr 17 04:08 2/
   614400 Apr 17 04:20 3/
   585728 Apr 17 04:28 4/
   581632 Apr 17 04:40 5/
   602112 Apr 17 04:48 6/
   614400 Apr 17 04:56 7/
   585728 Apr 17 05:08 8/
   602112 Apr 17 05:16 9/
  3993995 Apr 17 05:21 assembly.fa
649644248 Apr 17 06:21 joinedreads.fastq
```

Before running, to create a Run folder and enter it.

```
mkdir Run && cd Run
```

To get high-quality and long-length reads using runGetFastq.py:

```
usage: runGetFastq.py [-h] inpath

positional arguments:
  inpath      the path to the barcoded folder (i.e. fast5)

optional arguments:
  -h, --help  show this help message and exit
```

runGetFastq.py ../albacore2.1.7/fast5/

You will get 12 barcode folders, each contains 40× long-length reads with quality higher than that in the first quantile (readsA.fastq), 40× high-quality reads with length longer than that in the first quantile (readsB.fastq) and the concatenated reads (A+B reads: reads.fastq). The distributions of read quality and read length can be seen in Supplementary Figure 3, the 40× A reads and the 40× B reads are shown in red and blue points, respectively.

```
  9635916 Apr 19 03:49 barcode01.txt
  3450582 Apr 19 03:49 barcode01.txtA
  6185405 Apr 19 03:49 barcode01.txtB
606559130 Apr 19 03:49 reads.fastq
322340424 Apr 19 03:49 readsA.fastq
284218706 Apr 19 03:49 readsB.fastq
```

To get miniasm assemblies using runmini.py

```
usage: runmini.py [-h]

Please run this in the Run folder!

optional arguments:
  -h, --help  show this help message and exit
```

runmini.py

```
['barcode01']
barcode01, running minimap and miniasm......
  utg000001c_len=3820257
  utg000002c_len=72475
  utg000003c_len=90587
  utg000004c_len=7906
barcode01, running minimap and miniasm on A reads......
  utg000001l_len=3829530
  utg000002c_len=72316
  utg000003c_len=90544
barcode01, running minimap and miniasm on B reads......
  utg000001l_len=3291920
  utg000002l_len=90919
  utg000003l_len=403837
  utg000004l_len=101580
  utg000005l_len=30366
  utg000006l_len=27424
```

You will get 3 assemblies inside each barcode folder.

```
  3991318 Apr 19 06:15 assembly.fa
  3992461 Apr 19 06:15 assemblyA.fa
  3946188 Apr 19 06:15 assemblyB.fa
```

To run canu with the sampling strategy by using runAssembly.py:

```
usage: runAssembly.py [-h]

Please run this in the Run folder, and make sure run runmini.py in advance!

optional arguments:
  -h, --help  show this help message and exit
```

runAssembly.py

```
[illegible]
['barcode01']
barcode01
Number of circular sequences in assembly.fa: 4
Number of circular sequences in assemblyA.fa: 2
Number of circular sequences in assemblyB.fa: 0
runcanu.py 3991318
canu -p canu -d canu. genomeSize=3991318 corOutCoverage=1000 -nanopore-raw reads.fastq gnuplotTested=true
-- Canu 1.6
--
-- CITATIONS
--
-- Koren S, Walenz BP, Berlin K, Miller JR, Phillippy AM.
-- Canu: scalable and accurate long-read assembly via adaptive k-mer weighting and repeat separation.
-- Genome Res. 2017 May;27(5):722-736.
[illegible]
```

As shown in the above process, with the runmini-assembled files, an estimated genome size is obtained and used by runAssembly.py to utilize Canu v1.6 for the subsequent assembly. In this case, Canu is used to assemble A+B reads (reads.fastq) to produce one assembly (Canu.). Then, Canu is used to assemble 40× corrected reads sampled from the corrected A+B reads five times. Each assembly produced by Canu (canu.contigs.fasta) is checked for circularity and the presence of zero depth in misassemblies by using Nucmer and GraphMap, respectively, to prepare a file containing circular and zero-depth-free contigs (cirseqN.fa). By comparing the file size of cirseqN.fa with that of the assembly (assembly.fa) obtained from runmini.py, the number of successful assemblies (*i.e.*, cirseqN.fa > 0.95*assembly.fa) is counted. In this case, the sampling strategy is performed for four times (canu.1~canu.4).

```
[illegible]
cat canu.*/cirseqN.fa > allcir.fa
RemoveSeq.py allcir.fa canu.cir.fa
Running nucmer......
{'Seq7_len=102151', 'Seq3_len=103355', 'Seq13_len=102520', 'Seq11_len=103868', 'Seq6_len=100052'}
{'Seq9_len=80038', 'Seq4_len=79783', 'Seq10_len=79963', 'Seq1_len=81157', 'Seq12_len=80651'}
{'Seq2_len=3848243', 'Seq8_len=3852502', 'Seq14_len=3846849'}
{'Seq15_len=12082', 'Seq5_len=11141'}
AddSeq.py assembly_concir.fa canu.cir.fa fpseq.fa
Adding A to B...
A assembly contains:
utg000003c_len=90587_C:1.000000
utg000002c_len=72475_C:1.000000
utg000001c_len=3820257_C:1.000000
utg000004c_len=7906_C:1.000000
B assembly contains:
Seq11_len=103868
Seq1_len=81157
Seq8_len=3852502
Seq15_len=12082
The maximum length of original assembly: 3852502
Running nucmer......
utg000002c_len=72475_C:1.000000 72710
   Seq11_len=103868 103868
   0.13802133477105558
   Seq1_len=81157 81157
   0.9990019345219759
   Seq8_len=3852502 3852502
   0.0005954571860053544
utg000001c_len=3820257_C:1.000000 3860097
   Seq11_len=103868 103868
   0.03582431547733662
   Seq1_len=81157 81157
   0.049299505895979395
   Seq8_len=3852502 3852502
   0.9999628812652142
utg000004c_len=7906_C:1.000000 8053
   Seq15_len=12082 12082
   0.9977652706505545
utg000003c_len=90587_C:1.000000 91019
   Seq11_len=103868 103868
   0.9996148958293218
   Seq1_len=81157 81157
   0.1386941360572717
   Seq8_len=3852502 3852502
   0.002450096067438771
```

As shown in the above process, all circular contigs produced by Canu are concatenated into a file named allcir.fa: cat canu.*/cirseqN.fa > allcir.fa. All-vs-all alignment of allcir.fa is performed using Nucmer to filter pair alignments between circular contigs with an alignment rate of ≥0.2, an aligned length of ≥2500 bp, and an identity of >0.98. The pair alignments are used as connected components in an undirected graph and then are analyzed using NetworkX (a Python package) to generate connected components in groups. In this case, there are four groups, and the longest length among each group is selected as a representative contig to form a representative assembly (canu.cir.fa). To compare circular sequences in miniasm assembly (assembly_concir.fa) with canu.cir.fa, representative contigs are produced (fpseq.fa): Addseq.py assembly_concir.fa canu.cir.fa fpseq.fa.

After running Assembly.py, you will get an assembly (fpseq.fa) ready for polishing.

ll barcode01/fpseq.fa

```
4049682 Apr 19 10:12 fpseq.fa
```

grep ‘>’ barcode01/fpseq.fa

```
>Seq8_len=3852502
>Seq1_len=81157
>Seq11_len=103868
>Seq15_len=12082
```

To run racon and nanopolish for consensus sequence generation using runConsensus.py:

```
usage: runConsensus.py [-h] f5path

Please run this in the Run folder!

positional arguments:
  f5path      the path to the barcoded folder (i.e. fast5)

optional arguments:
  -h, --help  show this help message and exit
```

runConsensus.py ../albacore2.1.7/fast5/

You will get a polished assembly (conseqs.fasta).

ll barcode01/conseqs.fasta

```
 4075999 Apr 22 04:17 conseqs.fasta
```

grep ‘>’ barcode01/conseqs.fasta

```
>Seq1_len=3877533
>Seq2_len=104598
>Seq3_len=81625
>Seq4_len=12172
```

To get circular genomes by using finalize.py:

```
usage: finalize.py [-h] outpath

Please run this in the Run folder!

positional arguments:
  outpath     the path to output

optional arguments:
  -h, --help  show this help message and exit
```

finalize.py ../results

```
To download dnaa nucleotides...
    iUP.py 100 800 'dnaa taxonomy:bacteria AND reviewed:yes' dnaa dnaa
To download repa nucleotides...
    iUP.py 100 800 'repa taxonomy:bacteria AND reviewed:yes' repa repa
['barcode01']
/Run/Run/barcode01
trimOverlapseq.py conseqs.fasta
fixstart.py trimmedseqs.fa
gc_skew based on 500-bp windows with a 20-bp sliding step along the sequences......
seqname: ['Seq4_len=12172', 'Seq1_len=3877533', 'Seq3_len=81625', 'Seq2_len=104598']
seqlen: [8076, 3865048, 72914, 91210]
Origin: [2090, 2975950, 67110, 46250]
Terminus: [7570, 1010170, 15350, 9950]
Running promer for searching the location of dnaA/repA, then for reverse complement seqs......
seqname: ['Seq1_len=3877533', 'Seq2_len=104598', 'Seq4_len=12172', 'Seq3_len=81625']
seqlen: [3865048, 91210, 8076, 72914]
Origin: [2975950, 46250, 2090, 67110]
Terminus: [1010170, 9950, 7570, 15350]
Running promer for searching the location of dnaA/repA and fixing start......
      85      381  |  2976580  2976876  |      297      297  |    88.89    93.94     0.00  |  1  1  sp|B0VMK0|DNAA_ACIBS        Seq1_len=3877533

for Seq1_len=3877533
dnaA/repA locates in 2976496:2976876
origin was corrected to 2976496 based on the start poistion of dnaA/repA
    fixing strat with 'ATG'
    bases were shifted by 1
renamefa.py startfixed.fa startfixed.contigs.fa
minimap2 -x map-ont -a -t 32 startfixed.contigs.fa reads.fastq | samtools view -T startfixed.contigs.fa -bS - | samtools sort -T long.bwa -o long.bam -
samtools index long.bam
```

In the above process, the redundant ends of consensus sequences (conseqs.fasta) are trimmed and the circular sequences are rearranged to begin at *dnaA*/*repA* or a position with the minimum value of the GC skew.

You will get an output folder (results) which contains barcode folders. Each barcode folder has a final assembly (startfixed.contigs.fa) along with its alignment files (long.bam*), reads.fastq and fpseq.fa. Please note that the sequencing reads we submitted to the NCBI (SRA accessions in Table 1) were 80× A+B reads (reads.fastq) not the basecalled reads (joinedreads.fastq).

```
  4049682 Apr 22 06:38 fpseq.fa
326842339 Apr 22 06:38 long.bam
    88648 Apr 22 06:38 long.bam.bai
606559130 Apr 22 06:38 reads.fastq
  4037317 Apr 22 06:38 startfixed.contigs.fa
```

grep '>' startfixed.contigs.fa

```
>Seq1_len=3865048
>Seq2_len=91210
>Seq3_len=72914
>Seq4_len=8076
```

The sequences can be manually examined with Tablet to confirm the uniformity and continuity of sequencing coverage:

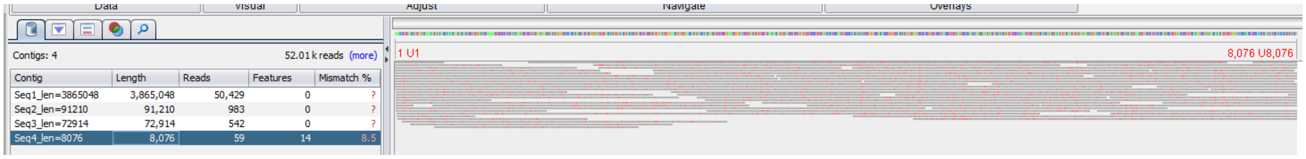

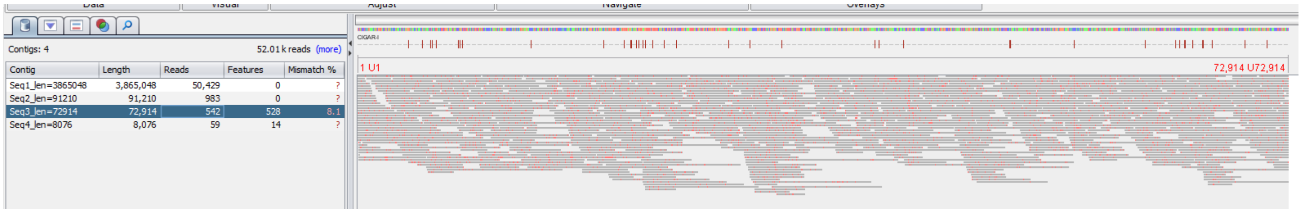

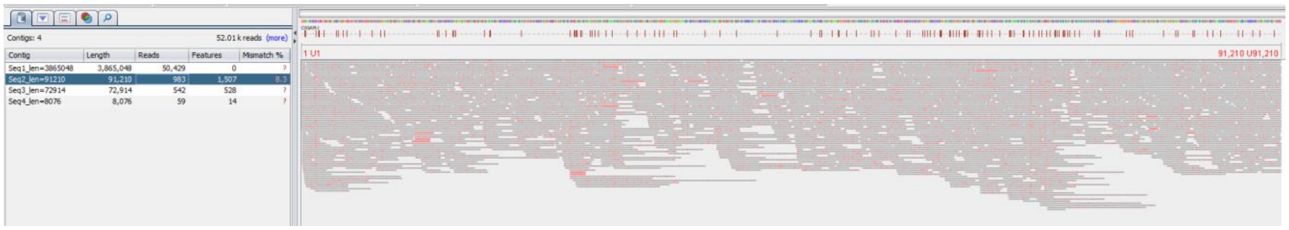

To get 40× long-length reads without considering quality (40× A* reads):

```
vi /opt/CCBGpipe/CCBGpipe/runGetFastq.py
```

amount=int(gsize)*40

comm='GetFastqA.py -i {0}.txt -q {0}/ -t {1} -o {2}/{0}/'.format(i,amount,cwd)

```
mkdir Run_40X_A && cd Run_40X_A
```

```
runGetFastq.py ../albacore2.1.7/fast5/
```

To get 40× high-quality reads without considering length (40× B* reads):

```
vi /opt/CCBGpipe/CCBGpipe/runGetFastq.py
```

amount=int(gsize)*40

comm='GetFastqB.py -i {0}.txt -q {0}/ -t {1} -o {2}/{0}/'.format(i,amount,cwd)

```
mkdir Run_40X_B && cd Run_40X_B
```

```
runGetFastq.py ../albacore2.1.7/fast5/
```

Comparison of assemblers

To compare long-read assemblers, we have conducted Canu (v1.7), Flye (2.3.3-g47cdd0b), HINGE, and miniasm (0.2-r168-dirty) to assemble the sequencing reads (joinedreads.fastq) of 12 barcodes produced by extract.py. Genome size and number of contigs produced by these assemblers are listed below:

	Canu1.7	Flye	HINGE*	Miniasm
Barcode01	4,052,016 (4)	4,100,783 (4)	7,886,155 (4)	4,000,072 (4)
Barcode02	4,335,522 (5)	4,376,632 (4)	8,434,591 (6)	4,280,305 (5)
Barcode03	4,169,271 (1)	4,255,666 (1)	8,332,632 (2)	4,135,056 (1)
Barcode04	4,178,606 (5)	4,170,853 (5)	7,785,453 (2)	4,051,959 (5)
Barcode05	3,890,082 (6)	3,964,936 (3)	7,769,065 (8)	3,857,283 (5)
Barcode06	4,284,616 (4)	4,349,745 (4)	8,367,626 (6)	4,236,313 (4)
Barcode07	4,603,999 (12)	4,555,369 (6)	8,682,983 (12)	4,437,000 (7)
Barcode08	4,313,070 (9)	4,386,356 (6)	8,298,512 (6)	4,339,396 (9)
Barcode09	2,923,777 (3)	2,963,344 (2)	5,841,113 (4)	2,901,692 (3)
Barcode10	2,951,690 (6)	2,946,201 (3)	5,798,100 (4)	2,892,865 (3)
Barcode11	2,895,086 (3)	2,961,146 (2)	5,814,014 (4)	2,895,471 (4)
Barcode12	3,046,048 (7)	3,022,955 (3)	5,889,033 (4)	2,910,091 (2)

*Please note that HINGE output sequences and their reverse complement sequences as well. Therefore, the corresponding genome size and the number of contigs are half the values listed in the above table.

Although Canu outputs "suggestCircular=yes" in the header line for circular sequences, we examined circularity ourselves.

grep '>' barcode01/canu/canu.contigs.fasta

```
>tig00000001 len=3852774 reads=14175 covStat=5237.52 gappedBases=no class=contig suggestRepeat=no suggestCircular=yes
>tig00000045 len=81189 reads=104 covStat=206.36 gappedBases=no class=contig suggestRepeat=no suggestCircular=yes
>tig00000046 len=104045 reads=169 covStat=236.35 gappedBases=no class=contig suggestRepeat=no suggestCircular=yes
>tig00000048 len=14008 reads=5 covStat=21.13 gappedBases=no class=contig suggestRepeat=no suggestCircular=no
```

CheckCirN.py barcode01/canu/canu.contigs.fasta

```
tig00000001 with 3852774 bp is circular
tig00000045 with 81189 bp is circular
tig00000048 with 14008 bp is circular
tig00000046 with 104045 bp is circular
```

Please note that contigs in an assembly produced by Canu are sometimes redundant, we take barcode10 as an example:

grep '>' barcode10/canu/canu.contigs.fasta

```
>tig00000012 len=5724 reads=92 covStat=-56.98 gappedBases=no class=contig suggestRepeat=no suggestCircular=no
>tig00000023 len=2859415 reads=7874 covStat=3361.99 gappedBases=no class=contig suggestRepeat=no suggestCircular=no
>tig00001008 len=20623 reads=1 covStat=0.00 gappedBases=no class=contig suggestRepeat=no suggestCircular=no
>tig00001009 len=25679 reads=33 covStat=13.83 gappedBases=no class=contig suggestRepeat=no suggestCircular=no
>tig00001010 len=23814 reads=23 covStat=31.11 gappedBases=no class=contig suggestRepeat=no suggestCircular=no
>tig00001011 len=16435 reads=1 covStat=0.00 gappedBases=no class=contig suggestRepeat=no suggestCircular=no
```

We have confirmed that two contigs (tig00000023 and tig00000012) are circular (tig00000023 is chromosome and tig00000012 is the small plasmid with length of 3 Kbp), and two contigs (tig00001008 and tig00001011) should be discarded because they are orphan sequences (reads=1) and we have confirmed that they are partial sequences of 27-Kbp plasmid and chromosome respectively (as shown in the following figure).

```
tig00000023 with 2859415 bp is circular
tig00000012 with 5724 bp is circular
```

The sequence of tig00001009 is likely to be the missing plasmid of length 27 Kbp, but is partial. However, *the sequence of tig00001010 is redundant and it is part of chromosomal sequence* (tig00000023):

Sequences producing significant alignments:

Select: All None Selected:0

Alignments Download Graphics Distance tree of results

	Description	Max Score	Total Score	Query Cover	E value	Per. Ident	Accession
	tig00000023 len=2859415 reads=7874 covStat=3361.99 gappedBases=no class=contig suggestRepeat=no suggestCircular=no	35622	1.591e+05	100%	0.0	99.68%	Query_49369

In Flye output, there is a file named assembly_info.txt. We take barcode01 and barcode10 as examples:

seq_name	length	cov.	circ.	repeat	mult.	graph_path	
contig_1	3936731		79	+	-	1	1
contig_2	74243	43	+	-	1	2	
contig_3	81619	55	-	-	1	*,3,*	
contig_4	8190	22	+	+	1	4	

This file suggests that Flye produce 4 contigs for barcode01, and three are circular sequences. Contig_1, contig_2 and contig_4 represent one chromosome, one large plasmid and one small plasmid. However, *contig_3 is the partial sequence of the plasmid* with length of 91 Kbp.

seq_name	length	cov.	circ.	repeat	mult.	graph_path	
contig_1	2924831		218	+	-	1	1
contig_2	20184	159	-	+	1	2	
contig_3	1186	11	-	+	1	3	

This file suggests that Flye produce 3 contigs for barcode10, and only one contig (chromosome) is a circular sequence. Contig_2 and contig_3 represent *partial sequences of the two plasmids*.

Please note that miniasm outputs an assembly graph containing unitigs with “c” and “l” suffixes to represent circular and linear sequences, respectively. We take barcode01, barcode03 and barcode10 as examples:

```
grep '>' barcode01/assembly.fa
```

```
>utg000001c_len=3828949
>utg000002c_len=90854
>utg000003c_len=72332
>utg000004c_len=7937
```

This information suggests that miniasm produce 4 circular sequences for barcode01.

```
grep '>' barcode03/assembly.fa
```

```
>utg000001l_len=4135056
```

This information suggests that miniasm produce 1 liner sequence for barcode03.

```
grep '>' barcode10/assembly.fa
```

```
>utg000001l_len=2863013
>utg000002c_len=26736
>utg000003c_len=3116
```

This information suggests that miniasm produce 1 linear and 2 circular sequences for barcode10.

To reduce the complexity of comparisons, we show schematic relationships between assemblies and final release assemblies for barcode01 and barcode10, as below:

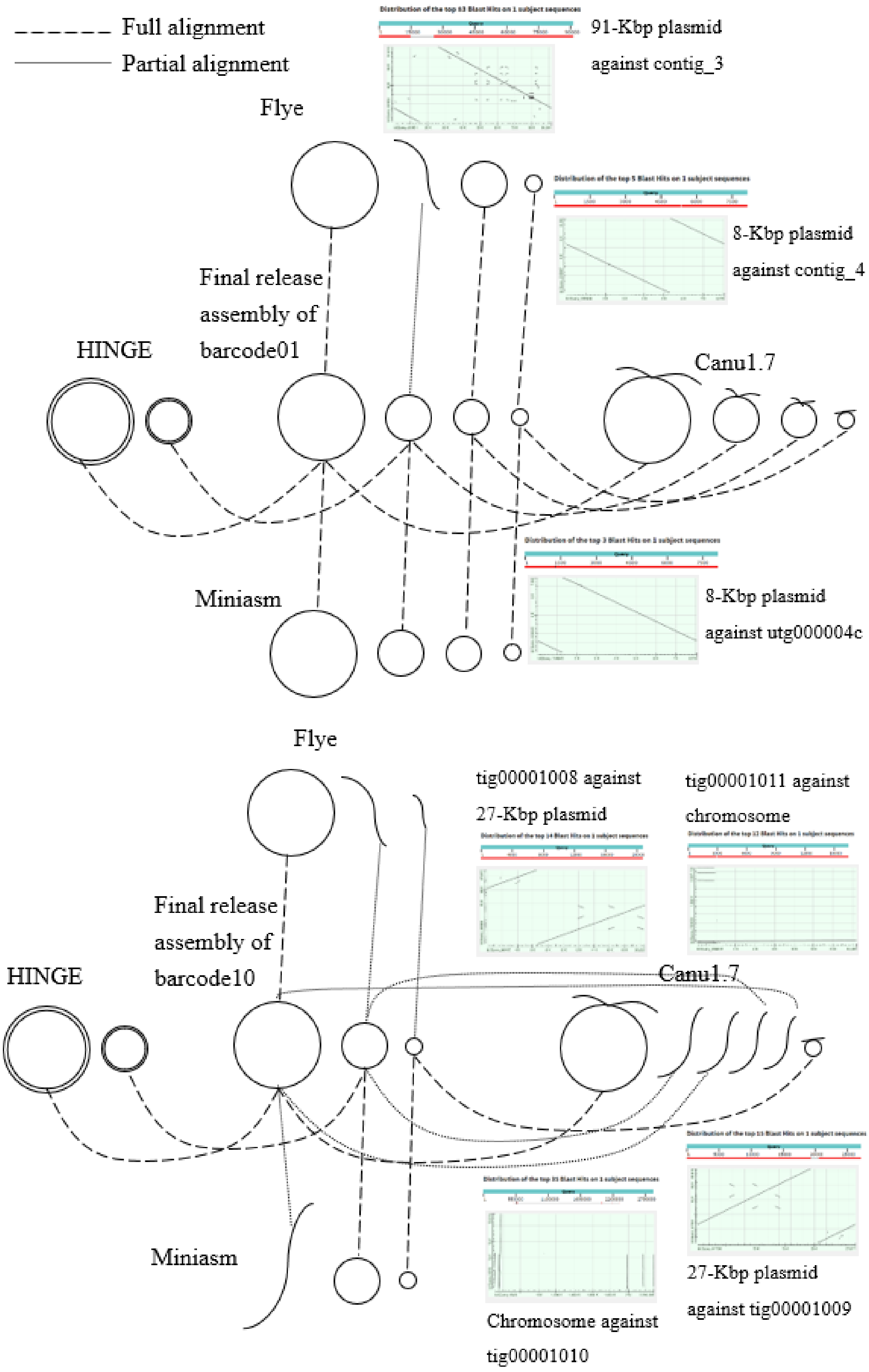
Full alignment
Partial alignment
91-Kbp plasmid
against contig_3
Flye
8-Kbp plasmid
against contig_4
Final release
assembly of
barcode01
HINGE
Canu1.7
Miniasm
8-Kbp plasmid
against utg000004c
Flye
tig00001008 against
27-Kbp plasmid
tig00001011 against
chromosome
Final release
assembly of
barcode10
HINGE
Canu1.7
Miniasm
27-Kbp plasmid
against tig00001009
Chromosome against
tig00001010

Besides, runtime of these assemblers is shown below:

	Canu1.7	Flye	HINGE	Miniasm
Barcode01	1 h 2 m 39 s	34 m 25 s	33 m 4 s	1 m 38 s
Barcode02	2 h 53 m 17 s	1 h 1 m 7 s	1 h 22 m 55 s	3 m 34 s
Barcode03	1 h 30 m 19 s	1 h 3 m 11 s	1 h 2 m 15 s	2 m 58 s
Barcode04	1 h 50 m 21 s	53 m 25 s	52 m 51 s	1 m 33 s
Barcode05	1 h 20 m 23 s	48 m 53 s	53 m 18 s	2 m 40 s
Barcode06	3 h 22 m 1 s	1 h 9 m 31 s	1 h 39 m 58 s	4 m 17 s
Barcode07	1 h 13 m 30 s	34 m 53 s	31 m 11 s	1 m 40 s
Barcode08	1 h 30 m 3 s	38 m 57 s	40 m 7 s	2 m 4 s
Barcode09	1 h 15 m 2 s	1 h 27 m 39 s	2 h 24 m 57 s	5 m 37 s
Barcode10	1 h 12 m 57 s	1 h 12 m 58 s	1 h 59 m 21 s	5 m 24 s
Barcode11	1 h 12 m 17 s	59 m 22 s	1 h 38 m 35 s	4 m 36 s
Barcode12	1 h 5 m 3 s	45 m 25 s	1 h 1 m 31 s	3 m 18 s

Canu1.7 command:

```
canu -p canu -d canu. genomeSize=gsize corOutCoverage=1000 -nanopore-raw reads.fastq
gnuplotTested=true
```

Please note that gsize was estimated based on the file size of Miniasm’s assembly

Flye command:

```
flye --nano-raw joinedreads.fastq --out-dir Flye_out --genome-size gsize --threads 32
```

Please note that gsize was estimated based on the file size of Miniasm’s assembly

HINGE command:

```
mkdir hingerun
cd hingerun
mv ../joinedreads.fastq ./
seqtk seq -a joinedreads.fastq > reads.fasta
hinge correct-head reads.fasta reads.np.fasta map.txt
fasta2DB barcode reads.np.fasta
DBsplit barcode
HPC.daligner -t32 barcode | bash -v
LAmerge barcode.las barcode.[0-9].las
DASqv -c100 barcode barcode.las
hinge filter --db barcode --las barcode --mlas -x barcode --config /opt/HINGE/utils/nominal.ini
hinge maximal --db barcode --las barcode --mlas -x barcode --config /opt/HINGE/utils/nominal.ini
hinge layout --db barcode --las barcode -x barcode --config /opt/HINGE/utils/nominal.ini -o barcode
hinge clip-nanopore barcode.edges.hinges barcode.hinge.list myRun
hinge draft-path ./ barcode barcodemyRun.G2.graphml
hinge draft --db barcode --las barcode.las --prefix barcode --config /opt/HINGE/utils/nominal.ini --
out barcode.draft
hinge correct-head barcode.draft.fasta barcode.draft.np.fasta draft_map.txt
fasta2DB draft barcode.draft.np.fasta
HPC.daligner -t32 barcode draft | bash -v
hinge consensus draft barcode draft.barcode.las barcode.consensus.fasta
/opt/HINGE/utils/nominal.ini
```

```
hinge gfa ./ barcode barcode.consensus.fasta
cd ..
```

Miniasm command:

```
minimap2 -x ava-ont -t50 joinedreads.fastq joinedreads.fastq > mapreads.paf
miniasm -f joinedreads.fastq mapreads.paf > assembly.gfa
getfa.py assembly.gfa
rm mapreads.paf assembly.gfa
```

Applying CCBGpipe to assemble *Klebsiella pneumoniae*

To validate the completeness of assemblies produced by CCBGpipe, we have run CCBGpipe for 7 samples (their depth of coverage of ONT reads >80×). Please note that the reason why we did not use all the 12 samples is that the depth for the other five samples is quite low (<50×). Because Wick et al have subsampled the Nanopore sequencing down to 500 Mbp high quality reads, and they did not provide the raw fast5 file, we decided to use the high-quality reads. These subsampled ONT reads were downloaded from https://figshare.com/articles/Subsampled_ONT_reads/5171491.

prepare_kp.py

You will get an output folder (/Run/5171491_kp) to contain the seven sequencing reads:

cd /Run/5171491_kp

```
/Run/5171491_kp# ll */reads.fastq
oot root 1001818676 Feb 11 17:24 barcode01/reads.fastq
oot root 1001655936 Feb 11 17:24 barcode02/reads.fastq
oot root  930151438 Feb 11 17:24 barcode06/reads.fastq
oot root 1002011724 Feb 11 17:24 barcode07/reads.fastq
oot root  889721000 Feb 11 17:24 barcode08/reads.fastq
oot root 1001605690 Feb 11 17:24 barcode10/reads.fastq
oot root 1001520910 Feb 11 17:24 barcode12/reads.fastq
```

runmini.py

```
['barcode12', 'barcode07', 'barcode01', 'barcode06', 'barcode02', 'barcode08', 'barcode10']
barcode01, running minimap and miniasm......
  utg000001c_len=5236334
  utg000002c_len=108082
  utg000003c_len=70413


barcode02, running minimap and miniasm......
  utg000001c_len=5244603
  utg000002c_len=109389
  utg000003c_len=70273


barcode06, running minimap and miniasm......
  utg000001c_len=5241224
  utg000002c_len=238830
  utg000003c_len=69849


barcode07, running minimap and miniasm......
  utg000001c_len=5217086
  utg000002c_len=110602
  utg000003c_len=162957


barcode08, running minimap and miniasm......
  utg000001c_len=5246200


barcode10, running minimap and miniasm......
  utg000001c_len=5145920
  utg000002c_len=249829
  utg000003l_len=155433
  utg000004c_len=108746
  utg000005c_len=127910
  utg000006c_len=5673
```

```
barcode12, running minimap and miniasm......
  utg000001c_len=5121992
  utg000002c_len=306769
  utg000003c_len=224802
  utg000004c_len=139521
  utg000005c_len=96832
  utg000006l_len=4516
  utg000007c_len=2813
  utg000008c_len=6873
  utg000009c_len=5472
  utg000010l_len=5012
  utg000011c_len=3287
```

After running runmini.py, you will get miniasm assemblies (assembly.fa). Miniasm outputs an assembly graph containing unitigs with "c" and "l" suffixes to represent circular and linear sequences, respectively. The number circular contigs can be obtained accordingly.

runAssembly.py

After running runAssembly.py, you will get assemblies ready for polishing (fpseq.fa) for each barcode.

Nanopolish requires fast5 for sequence consensus, but that were not provided from the website. We therefore **did not perform sequence consensus**. To compare the numbers of circular contigs (assembly.fa and fpseq.fa) with that obtained from https://github.com/rrwick/Bacterial-genome-assemblies-with-multiplex-MinION-sequencing/blob/master/results.xlsx:

	Canu*	Unicycler*	Miniasm	Uni-hybrid*	CCBGpipe	Complete*
Barcode01	2	**3**	**3**	**3**	**3**	3
Barcode02	2	**3**	**3**	**3**	**3**	3
Barcode06	2	3	3	6	5	7
Barcode07	**3**	**3**	**3**	**3**	**3**	3
Barcode08	0	0	**1**	**1**	**1**	1
Barcode10	2	7	5	6	7	8
Barcode12	5	7	9	**11**	**11**	11
Fraction	16/36 (44.4%)	26/36 (72.2%)	27/36 (75%)	**33/36 (91.7%)**	**33/36 (91.7%)**	36/36 (100%)

* The number of circular contigs for Canu, Unicycler, Uni-hybrid and Complete are excerpted from the results (https://github.com/rrwick/Bacterial-genome-assemblies-with-multiplex-MinION-sequencing/blob/master/results.xlsx). Canu: Canu long-read-only assemblies; Unicycler: Unicycler long-read-only assemblies; Uni-hybrid: Unicycler hybrid assemblies; Complete: Manually completed assemblies.

Applying CCBGpipe to assemble *Staphylococcus aureus*

We have two *S. aureus* strains (M013 and VGC1). Each of them has been sequenced with both Illumina and Oxford Nanopore technologies. We therefore used Unicycler to hybrid assemble Illumina reads with MinION long reads and took the assemblies as reference genomes.

unicycler -1 R1.fastq -2 R2.fastq -l joinedreads.fastq -o reference

unicycler -l joinedreads.fastq -o unicycler

We then performed quality assessment using Quast (http://quast.bioinf.spbau.ru/):

M013 (2 circular sequences)

Worst Median Best ☑ Show heatmap

Genome statistics	startfixed.contigs	assembly
Genome fraction (%)	100	100
Duplication ratio	1	0.999
Largest alignment	2 794 945	2 792 590
Total aligned length	2 816 631	2 814 234
NGA50	2 794 945	2 792 590
LGA50	1	1
**Misassemblies**		
# misassemblies	0	0
Misassembled contigs length	0	0
**Mismatches**		
# mismatches per 100 kbp	4.47	24.46
# indels per 100 kbp	110.59	268.08
# N's per 100 kbp	0	0
**Statistics without reference**		
# contigs	2	2
Largest contig	2 794 952	2 792 590
Total length	2 816 639	2 814 237
Total length (>= 1000 bp)	2 816 639	2 814 237
Total length (>= 10000 bp)	2 816 639	2 814 237
Total length (>= 50000 bp)	2 794 952	2 792 590

VGC1 (3 circular sequences)

Worst Median Best ☑ Show heatmap

Genome statistics	startfixed.contigs	assembly
Genome fraction (%)	100	99.847
Duplication ratio	1.001	0.999
Largest alignment	2 858 827	2 386 621
Total aligned length	2 891 198	2 883 057
NGA50	2 858 827	2 386 621
LGA50	1	1
**Misassemblies**		
# misassemblies	0	1
Misassembled contigs length	0	2 853 674
**Mismatches**		
# mismatches per 100 kbp	4.5	22.88
# indels per 100 kbp	131.55	258.16
# N's per 100 kbp	0	0
**Statistics without reference**		
# contigs	3	2
Largest contig	2 858 827	2 853 674
Total length	2 891 198	2 883 057
Total length (>= 1000 bp)	2 891 198	2 883 057
Total length (>= 10000 bp)	2 888 193	2 883 057
Total length (>= 50000 bp)	2 858 827	2 853 674

As shown in the above results, our pipeline (CCBGpipe) produce complete (5 circular sequences) and accurate (>99.8%) assemblies in comparision to the long-read-only Unicycler assemblies (4 circular sequences) with accurace of 99.7%. By comparing the MinION reads (reads in joinedreads.fastq) to the reference genome assembled by hybrid unicycler using blastn, approximately 90% sequence identity is obtained, which suggests that the accuracy of raw MinION reads is around 90%. In running CCBGpipe, representative contigs (fpseq.fa) assembled by Canu are produced. The Canu-assembled assemblies have accuracy of 99.5%.

Worst Median Best ☑ Show heatmap

Genome statistics	startfixed.contigs	fpseq	assembly
Genome fraction (%)	100	99.956	100
Duplication ratio	1	1.004	0.999
Largest alignment	2 794 945	2 796 129	2 792 590
Total aligned length	2 816 631	2 825 809	2 814 234
NGA50	2 794 945	2 796 129	2 792 590
LGA50	1	1	1
**Misassemblies**			
# misassemblies	0	1	0
Misassembled contigs length	0	29 680	0
**Mismatches**			
# mismatches per 100 kbp	4.47	9.27	24.46
# indels per 100 kbp	110.59	583.17	268.08
# N's per 100 kbp	0	0	0
**Statistics without reference**			
# contigs	2	2	2
Largest contig	2 794 952	2 796 497	2 792 590
Total length	2 816 639	2 826 177	2 814 237
Total length (>= 1000 bp)	2 816 639	2 826 177	2 814 237
Total length (>= 10000 bp)	2 816 639	2 826 177	2 814 237
Total length (>= 50000 bp)	2 794 952	2 796 497	2 792 590

Worst Median Best ☑ Show heatmap

Genome statistics	startfixed.contigs	fpseq	assembly
Genome fraction (%)	100	99.995	99.847
Duplication ratio	1.001	1.005	0.999
Largest alignment	2 858 827	2 854 824	2 386 621
Total aligned length	2 891 198	2 904 279	2 883 057
NGA50	2 858 827	2 854 824	2 386 621
LGA50	1	1	1
**Misassemblies**			
# misassemblies	0	3	1
Misassembled contigs length	0	49 535	2 853 674
**Mismatches**			
# mismatches per 100 kbp	4.5	6.09	22.88
# indels per 100 kbp	131.55	490.91	258.16
# N's per 100 kbp	0	0	0
**Statistics without reference**			
# contigs	3	3	2
Largest contig	2 858 827	2 854 911	2 853 674
Total length	2 891 198	2 904 446	2 883 057
Total length (>= 1000 bp)	2 891 198	2 904 446	2 883 057
Total length (>= 10000 bp)	2 888 193	2 899 121	2 883 057
Total length (>= 50000 bp)	2 858 827	2 854 911	2 853 674

So, to summarize the accuracies for reads, long-read-only Unicycler assemblies, Canu assemblies and CCBGpipe assemblies are listed:

Sequence	raw MinION reads	Unicycler	Canu	CCBGpipe
File name	joinedreads.fastq	assembly.fasta	fpseq.fa	startfixed.contigs.fa
Accuracy (%)	90	99.7	99.5	99.87

Basecalling with Guppy

Oxford Nanopore Technologies released Guppy to replace Albacore in December 24, 2018. The fast5 file format has been changed from "one fast5 file per read" to "multi-read fast5" file format. Guppy can handle multi-read fast5 files.

```
Usage:

With config file:
  guppy_basecaller -i <input path> -s <save path> -c <config file> [options]
With flowcell and kit name:
  guppy_basecaller -i <input path> -s <save path> --flowcell <flowcell name>
    --kit <kit name>
List supported flowcells and kits:
  guppy_basecaller --print_workflows
Use server for basecalling:
  guppy_basecaller -i <input path> -s <save path> -c <config file>
    --port <server address> [options]
```

guppy_basecaller -i Fast5 -s guppy_out

After running guppy_basecaller, you will get an output folder (e.g., guppy_out) to contain fastq_runid_*.fastq, guppy_basecaller_log-*.log and a file named sequencing_summary.txt.

```
    5242791 Feb 27 16:34 guppy_basecaller_log-2019-02-27_16-34-48.log*
   45375809 Feb 27 16:29 fastq_runid_692c3fc8e660735e591891d011e8252dd773c86a_3.fastq*
   45697271 Feb 27 16:29 fastq_runid_692c3fc8e660735e591891d011e8252dd773c86a_1.fastq*
   43802554 Feb 27 16:29 fastq_runid_692c3fc8e660735e591891d011e8252dd773c86a_2.fastq*
   46852903 Feb 27 16:29 fastq_runid_692c3fc8e660735e591891d011e8252dd773c86a_0.fastq*
  335501159 Feb 27 16:29 sequencing_summary.txt*
    5242725 Feb 27 16:29 guppy_basecaller_log-2019-02-27_16-29-36.log*
```

```
   3688243 Feb 28 00:18 sequencing_telemetry.js*
   7989237 Feb 28 00:16 fastq_runid_9997ff79d58a080a9f9c8f5a623494b850f1a65c_29.fastq*
   7807916 Feb 28 00:16 fastq_runid_9997ff79d58a080a9f9c8f5a623494b850f1a65c_28.fastq*
   7480075 Feb 28 00:15 fastq_runid_692c3fc8e660735e591891d011e8252dd773c86a_316.fastq*
  18523864 Feb 28 00:13 fastq_runid_692c3fc8e660735e591891d011e8252dd773c86a_315.fastq*
  23645573 Feb 28 00:13 fastq_runid_692c3fc8e660735e591891d011e8252dd773c86a_314.fastq*
   4099731 Feb 28 00:13 guppy_basecaller_log-2019-02-28_00-13-02.log*
  42533852 Feb 28 00:09 fastq_runid_692c3fc8e660735e591891d011e8252dd773c86a_313.fastq*
  44625532 Feb 28 00:07 fastq_runid_692c3fc8e660735e591891d011e8252dd773c86a_312.fastq*
   5242874 Feb 28 00:06 guppy_basecaller_log-2019-02-28_00-06-56.log*
  45048531 Feb 28 00:06 fastq_runid_692c3fc8e660735e591891d011e8252dd773c86a_311.fastq*
  44823645 Feb 28 00:05 fastq_runid_692c3fc8e660735e591891d011e8252dd773c86a_310.fastq*
  45051656 Feb 28 00:04 fastq_runid_692c3fc8e660735e591891d011e8252dd773c86a_309.fastq*
   5242761 Feb 28 00:02 guppy_basecaller_log-2019-02-28_00-02-03.log*
  45305017 Feb 28 00:00 fastq_runid_692c3fc8e660735e591891d011e8252dd773c86a_308.fastq*
```

guppy_barcoder -i guppy_out -s barcoding

After running guppy_barcorder, you will get the barcode folders and a file named barcoding_summary.txt:

```
         0 Mar  6 17:40 barcode01/
         0 Mar  6 17:40 barcode02/
         0 Mar  6 17:40 barcode03/
         0 Mar  6 17:40 barcode04/
         0 Mar  6 17:40 barcode05/
         0 Mar  6 17:40 barcode06/
         0 Mar  6 17:40 barcode07/
         0 Mar  6 17:40 barcode08/
         0 Mar  6 17:40 barcode09/
         0 Mar  6 17:40 barcode10/
         0 Mar  6 17:40 barcode11/
         0 Mar  6 17:40 barcode12/
         0 Mar  6 17:40 barcode12a/
 381426165 Feb 28 08:51 barcoding_summary.txt*
```

Before running runGetFastq.py, you need to get barcode files and barcode folders using preprocess.py:

```
usage: preprocess.py [-h] [-b BARCODING_SUMMARY.TXT]
                     [-s SEQUENCING_SUMMARY.TXT] [-o OUTDIR]

optional arguments:
  -h, --help            show this help message and exit
  -b BARCODING_SUMMARY.TXT
                        the path to barcoding_summary.txt
  -s SEQUENCING_SUMMARY.TXT
                        the path to sequencing_summary.txt
  -o OUTDIR             the path to output
```

preprocess.py -b barcoding/barcoding_summay.txt -s guppy_out/sequencing_summary.txt -o outdir

You will get barcode folders and index files for each barcode. Each barcode folder contains a fastq file (joinedreads.fastq), a miniasm-assembled assembly (assembly.fa) and a tsv file for filter_reads.

```
       0 Apr 29 15:46 barcode01/
 8697967 Apr 29 15:46 barcode01.txt*
       0 Apr 29 15:49 barcode02/
 8964207 Apr 29 15:49 barcode02.txt*
       0 Apr 29 15:50 barcode03/
 5078536 Apr 29 15:50 barcode03.txt*
       0 Apr 29 15:52 barcode04/
 5479142 Apr 29 15:52 barcode04.txt*
       0 Apr 29 15:53 barcode05/
 3548422 Apr 29 15:53 barcode05.txt*
       0 Apr 29 15:56 barcode06/
 9953248 Apr 29 15:56 barcode06.txt*
       0 Apr 29 15:58 barcode07/
 5394324 Apr 29 15:58 barcode07.txt*
       0 Apr 29 16:01 barcode08/
 6937720 Apr 29 16:01 barcode08.txt*
       0 Apr 29 16:04 barcode09/
 4127745 Apr 29 16:04 barcode09.txt*
       0 Apr 29 16:05 barcode10/
 2052522 Apr 29 16:05 barcode10.txt*
       0 Apr 29 16:14 barcode11/
17399894 Apr 29 16:14 barcode11.txt*
       0 Apr 29 16:17 barcode12/
 6469695 Apr 29 16:17 barcode12.txt*
       0 Apr 29 16:17 barcode12a/
     980 Apr 29 16:17 barcode12a.txt*
```

cd outdir/barcode01 && ll

```
   5306782 Apr 29 16:25 assembly.fa*
   3167097 Apr 29 16:22 barcode01_readid.tsv*
1024997573 Apr 29 16:22 joinedreads.fastq*
```

With the above information, you can perform CCBGpipe by beginning with runGetFastq.py.

cd ../../

With the file listing read_id in readid.tsv, one can extract fast5 files using filter_reads (https://github.com/nanoporetech/fast5_research):

```
usage: filter_reads [-h] [--tsv_field TSV_FIELD] [--multi | --single]
                    [--prefix PREFIX] [--recursive] [--workers WORKERS]
                    input output filter

Extract reads from multi-read .fast5 files.

positional arguments:
  input                 Path to input multi-read .fast5 files.
  output                Output folder.
  filter                A .tsv file with column `read_id` defining required
                        reads.

optional arguments:
  -h, --help            show this help message and exit
  --tsv_field TSV_FIELD
                        Field name from `filter` file to obtain read IDs.
  --multi               Output multi-read files.
  --single              Output single-read files.
  --prefix PREFIX       Read file prefix.
  --recursive           Search recursively under `input` for source files.
  --workers WORKERS     Number of worker processes.
```

filter_reads --recursive --multi --workers 32 Fast5/ fast5/barcode01 outdir/barcode01/barcode01_readid.tsv

cd fast5/barcode01 && ll

```
430842128 Apr 29 16:37 mreads_file0.fast5*
416877652 Apr 29 16:38 mreads_file1.fast5*
449880516 Apr 29 16:50 mreads_file10.fast5*
456147998 Apr 29 16:51 mreads_file11.fast5*
488331623 Apr 29 16:52 mreads_file12.fast5*
487837632 Apr 29 16:53 mreads_file13.fast5*
434436888 Apr 29 16:55 mreads_file14.fast5*
429372355 Apr 29 16:56 mreads_file15.fast5*
428538948 Apr 29 16:57 mreads_file16.fast5*
481178711 Apr 29 16:58 mreads_file17.fast5*
440521711 Apr 29 17:00 mreads_file18.fast5*
431246831 Apr 29 17:01 mreads_file19.fast5*
444263740 Apr 29 16:39 mreads_file2.fast5*
473640816 Apr 29 17:02 mreads_file20.fast5*
185419829 Apr 29 17:03 mreads_file21.fast5*
413922684 Apr 29 16:41 mreads_file3.fast5*
443538757 Apr 29 16:42 mreads_file4.fast5*
473681704 Apr 29 16:43 mreads_file5.fast5*
456593379 Apr 29 16:44 mreads_file6.fast5*
425489286 Apr 29 16:46 mreads_file7.fast5*
460167083 Apr 29 16:47 mreads_file8.fast5*
435989594 Apr 29 16:48 mreads_file9.fast5*
```

2 Supplementary Figures

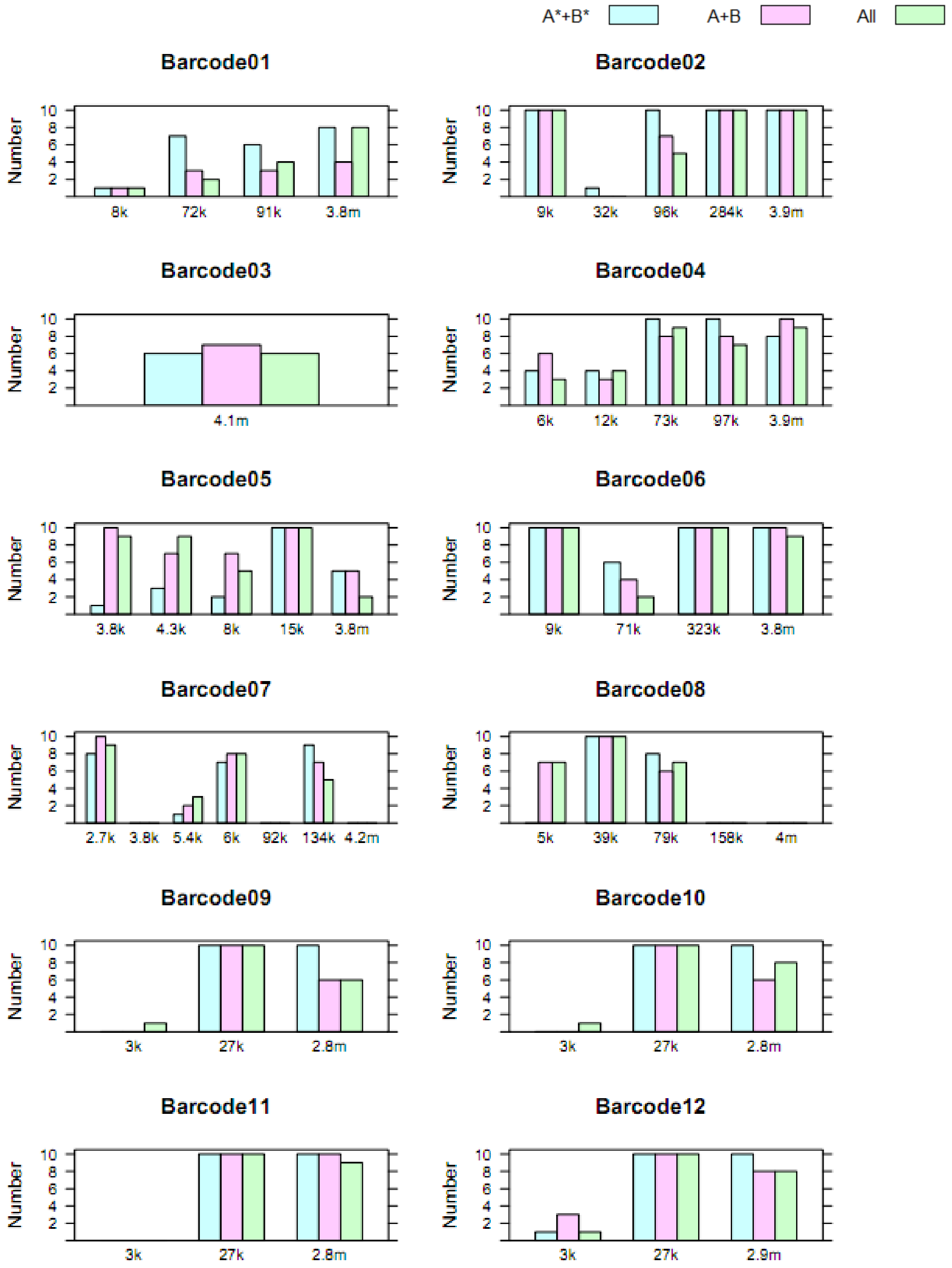

Supplementary Figure 1. Number of circular contigs produced by miniasm. Forty-fold sampling reads from three read sets (all reads, A*+B*, and A+B) were separately produced and assembled by miniasm ten times (y-axis) to see whether the 48 circular sequences (x-axis) were produced. A* and B* reads are the 40× reads ordered by length and quality, respectively, without considering the minimal quality and length, whereas A+B reads are the 40× long-length reads with quality higher than that in the first quantile plus the remaining 40× high-quality reads with length longer than that in the first quantile.

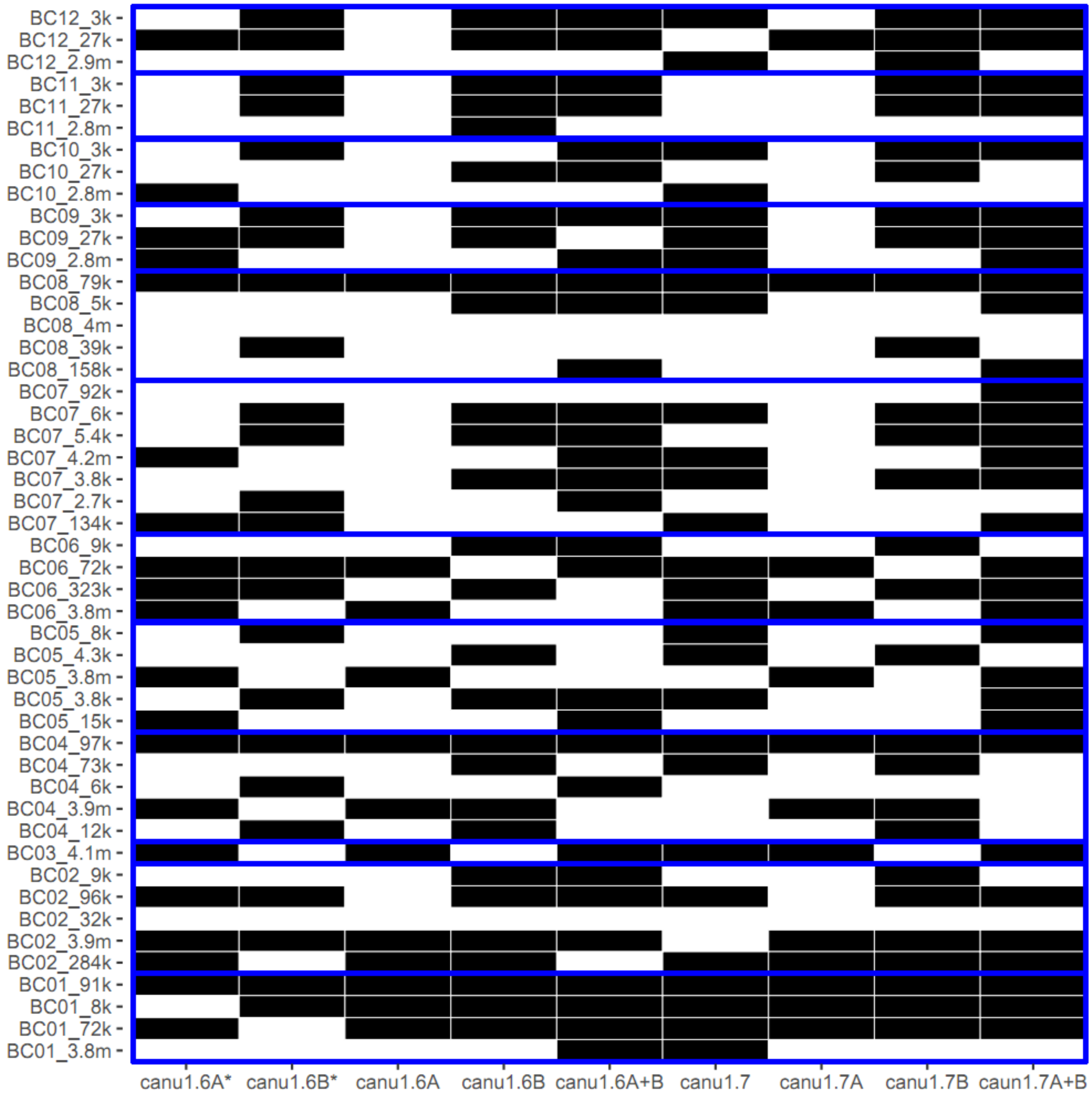

Supplementary Figure 2. Canu assemblies. Canu v1.6 (with parameters: corOutCoverage=1000 and nanopore-raw) was used to assemble 40× long-length reads (A*), 40× high-quality reads (B*), 40× long-length reads with quality higher than that in the first quantile (A), 40× high-quality reads with length longer than that in the first quantile (B), and A+B reads. Canu v1.7 was used to assemble all reads with default setting and to assemble A, B, and A+B reads with parameters: corOutCoverage=1000 and nanopore-raw. Filled bars represent circular contigs that were assembled.

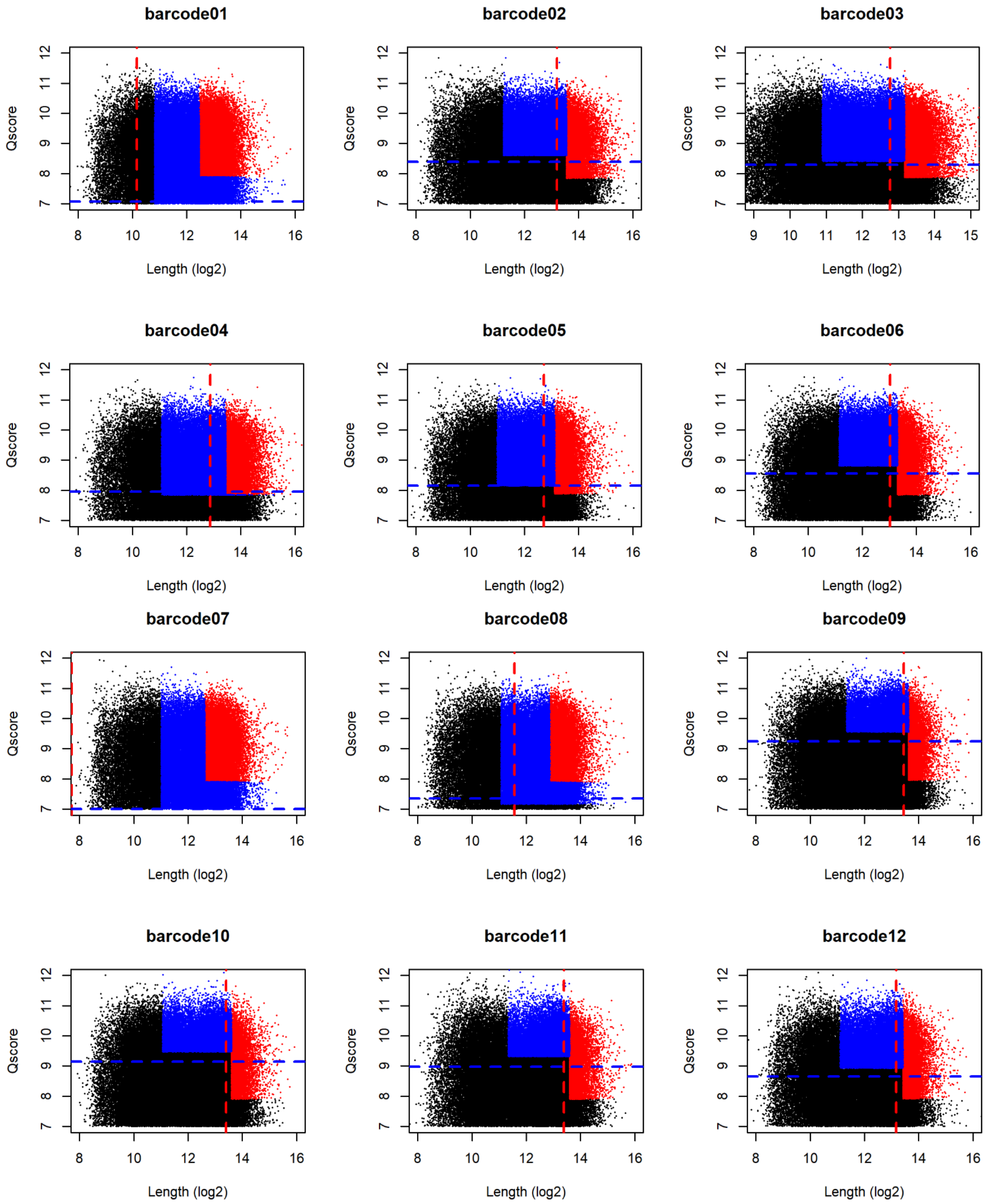

Supplementary Figure 3. Distributions of read quality (y-axis) and read length (x-axis, log2(read length)) for 80× long-length reads, high-quality reads, and A+B reads. Each dot denotes a sequencing read. The 80× long-length read set are the points right against the red dotted line, the 80× high-quality read set are the points above the blue dotted line, and A+B reads are the 40× long-length A reads with quality higher than that in the first quantile (red points) plus the remaining 40× high-quality B reads with length higher than that in the first quantile (blue points).
